# MRI-Guided Electrode Implantation for Chronic Intracerebral Recordings in a Rat Model of Post−Traumatic Epilepsy—Challenges and Gains

**DOI:** 10.3390/biomedicines10092295

**Published:** 2022-09-15

**Authors:** Xavier Ekolle Ndode-Ekane, Riikka Immonen, Elina Hämäläinen, Eppu Manninen, Pedro Andrade, Robert Ciszek, Tomi Paananen, Olli Gröhn, Asla Pitkänen

**Affiliations:** A.I. Virtanen Institute for Molecular Sciences, University of Eastern Finland, P.O. Box 1627, FI-70211 Kuopio, Finland

**Keywords:** cortical atrophy, hippocampal atrophy, intracerebral electrode, magnetic resonance imaging, posttraumatic epilepsy, traumatic brain injury

## Abstract

Brain atrophy induced by traumatic brain injury (TBI) progresses in parallel with epileptogenesis over time, and thus accurate placement of intracerebral electrodes to monitor seizure initiation and spread at the chronic postinjury phase is challenging. We evaluated in adult male Sprague Dawley rats whether adjusting atlas-based electrode coordinates on the basis of magnetic resonance imaging (MRI) increases electrode placement accuracy and the effect of chronic electrode implantations on TBI-induced brain atrophy. One group of rats (EEG cohort) was implanted with two intracortical (anterior and posterior) and a hippocampal electrode right after TBI to target coordinates calculated using a rat brain atlas. Another group (MRI cohort) was implanted with the same electrodes, but using T2-weighted MRI to adjust the planned atlas-based 3D coordinates of each electrode. Histological analysis revealed that the anterior cortical electrode was in the cortex in 83% (25% in targeted layer V) of the EEG cohort and 76% (31%) of the MRI cohort. The posterior cortical electrode was in the cortex in 40% of the EEG cohort and 60% of the MRI cohort. Without MRI-guided adjustment of electrode tip coordinates, 58% of the posterior cortical electrodes in the MRI cohort will be in the lesion cavity, as revealed by simulated electrode placement on histological images. The hippocampal electrode was accurately placed in 82% of the EEG cohort and 86% of the MRI cohort. Misplacement of intracortical electrodes related to their rostral shift due to TBI-induced cortical and hippocampal atrophy and caudal retraction of the brain, and was more severe ipsilaterally than contralaterally (*p* < 0.001). Total lesion area in cortical subfields targeted by the electrodes (primary somatosensory cortex, visual cortex) was similar between cohorts (*p* > 0.05). MRI-guided adjustment of coordinates for electrodes improved the success rate of intracortical electrode tip placement nearly to that at the acute postinjury phase (68% vs. 62%), particularly in the posterior brain, which exhibited the most severe postinjury atrophy. Overall, MRI-guided electrode implantation improved the quality and interpretation of the origin of EEG-recorded signals.

## 1. Introduction

Traumatic brain injury (TBI) is an alteration in brain function or brain pathology caused by an external force [1]. Postimpact secondary pathologies include axonal injury, neurodegeneration, blood–brain barrier dysfunction, and neuroinflammation, which can progress over days to weeks to years [2,3,4,5,6,7]. The progressive reorganization of neuronal networks can result in chronic comorbidities that compromise the quality of life, such as posttraumatic epilepsy (PTE) [8,9].

The risk of PTE increases with the severity of the TBI, and is approximately 16% after severe TBI [10]. The risk of epilepsy post-TBI increases with impact severity in both humans and animal models [11]. Clinical and experimental studies show that the seizure focus and epileptogenic network locate in cortical structures, including the cerebral cortex and hippocampus [12,13,14]. As epileptogenesis occurs over a period of weeks to months and the frequency of unprovoked seizures is low, mechanistic studies of the evolution of PTE require chronic electroencephalogram (EEG) recordings of the severely damaged brain with an ongoing progressive secondary pathology [11]. On the other hand, to detect unprovoked seizures (i.e., epilepsy diagnosis), electrodes are often implanted months to weeks after the injury. The usefulness of atlas coordinates for electrode placement into the atrophied cortical structures long after the occurrence of TBI and the effect of the presence of chronic intracerebral electrodes on brain pathology, however, are unknown.

In patients with epilepsy, advanced structural magnetic resonance imaging (MRI) techniques are used extensively in the clinic to facilitate intracerebral navigation during implantation of intracerebral EEG electrodes for identifying epileptic foci or for stimulation therapy [15,16]. To our knowledge, MRI guidance has not been applied in preclinical studies. One of the objectives of the National Institutes of Health (NIH)-funded Centers without Walls project EpiBioS4Rx (https://epibios.loni.usc.edu/; access on 14 March 2022) is to perform chronic intracerebral EEG recordings over the course of epileptogenesis after lateral fluid-percussion injury (FPI)-induced TBI. Lateral FPI is a widely used preclinical model of TBI, leading to epilepsy in approximately 25% of animals within 6 to 7 months postinjury [7,11,17,18]. Like in humans with PTE, progressive brain damage involves epileptogenic regions, including the cerebral cortex and hippocampus [7,19,20]. The progression of cortical and hippocampal atrophy and spatial distortion, however, varies significantly between animals over time [7,21,22,23].

Epileptogenesis studies often require large animal cohorts, and therefore optimizing the quality of EEG recordings and their interpretation in atrophied brain is critical for maintaining the feasibility and affordability of the studies [24]. As such, we aimed to develop methodologies to (a) maximize the accuracy of recording-electrode placement into the atrophied brain using preimplantation in vivo MRI, (b) reliably assess electrode locations to correctly interpret the origin of the EEG-recorded signal, and (c) respond to concerns related to the effect of chronic electrode implantations on TBI-induced brain atrophy. We hypothesized that (a) MRI-guided electrode implantation will improve the accuracy of perilesional intracortical and intrahippocampal electrode placements at a chronic postinjury time point, and (b) chronic electrode implantation does not worsen lateral FPI-induced cortical damage. The data presented include analysis of 297 electrode locations in 25 sham-operated experimental controls and 74 rats with severe TBI induced by lateral FPI in the University of Eastern Finland (UEF) subcohort of the EpiBioS4Rx Project 1. In 57 rats, electrode implantation was performed immediately after the injury (EEG cohort). In 42 rats, the electrodes were implanted after MRI was performed at 5 months postinjury (MRI cohort). At the end, all animals were perfused for ex vivo MRI and histology.

## 2. Materials and Methods

This study is part of the National Institutes of Health funded Centers without Walls international multicenter project EpiBioS4Rx, which aims to identify biomarkers for posttraumatic epileptogenesis (https://epibios.loni.usc.edu/, accessed 14 March 2022). We report data from the University of Eastern Finland (UEF) subcohort, including histological sections and MRI findings for assessing electrode locations.

### 2.1. Animals and Study Design

*Animals.* Adult male Sprague Dawley rats (300–350 g at the time of injury) were used. Animals were quarantined for 1 week (3–6 per cage) upon arrival at the animal facility. Thereafter, they were individually housed in a controlled environment (temperature 22 ± 1 °C; humidity 50–60%; lights on from 07:00 to 19:00 h) until the end of the experiments. Food and water were provided ad libitum for the duration of the study. All animal procedures were approved by the Animal Ethics Committee of the Provincial Government of Southern Finland and carried out in accordance with the guidelines of European Community Council Directives 2010/63/EU.

**Figure 1:**
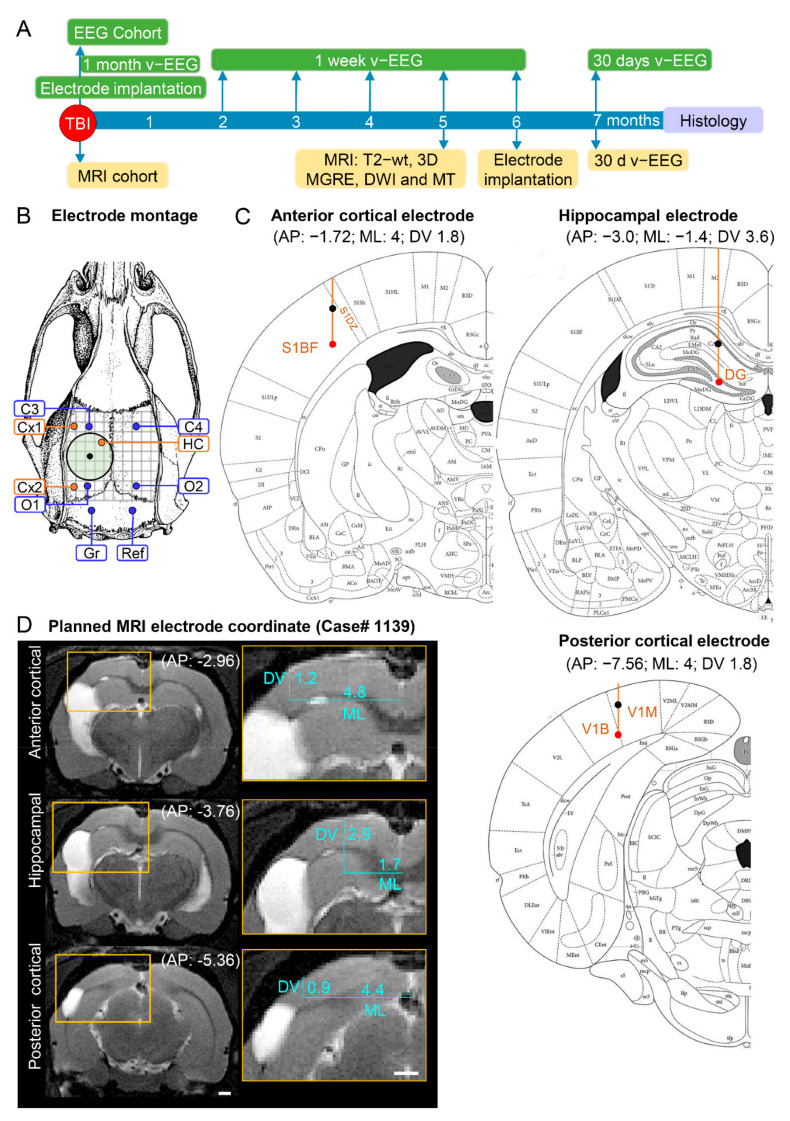
Study design, electrode montage, and atlas or MRI-planned electrode coordinates. (**A**) Study design. Following TBI, rats were divided into either the EEG or MRI cohort. The rats of the EEG cohort were implanted with electrodes after fully righting themselves following induction of TBI. The rats were followed up immediately afterward with 1 month video-EEG and then for 1 week monthly until the 6th post-TBI month. The rats of the MRI cohort were magnetic resonance-imaged at 5 months post-TBI and T2-wt images were used to calculate the coordinates of the intracerebral electrodes implanted at 6 months post-TBI. Both cohorts were continuously monitored with video-EEG for 30 days at 7 months post-TBI to diagnose epilepsy. At the end of the 7-month follow-up period, all rats were euthanized and the brains processed for histological identification of the location of the intracerebral electrodes. (**B**) Electrode montage used in the study. Four epidural screw electrodes (C3, C4, O1 and O2), 3 intracerebral bipolar wire electrodes (anterior cortical Cx1, posterior cortical Cx2, and hippocampal HC), a ground (Gr) and reference (Ref) electrode. (**C**) Atlas plates demonstrating the planned coordinates used in the EEG cohort to implant the anterior cortical, hippocampal, and posterior cortical electrodes. The black dot refers to the upper tip and the red dot to the lower tip of the bipolar electrode (1 mm apart). Reprinted/adapted with permission from [25]. 2007, Elsevier Inc. (**D**) MRI T2-wt images of rat 1139 demonstrating the planned-MRI coordinates of the intracerebral anterior cortical, hippocampal, and posterior cortical electrodes. The anteroposterior (AP) coordinate was determined by aligning the MR images with the atlas [25]. The mediolateral (ML) and dorsoventral (DV) coordinates were determined using ImageJ software (version 1.47v, Wayne Rasband and contributors, National Institute of Health, USA).

*Study design (*Figure 1*).* The study design, randomization, and interventions performed in the 2 study cohorts are summarized in Figure 1. The rats were divided into EEG (14 sham, 43 TBI) and MRI (11 sham, 31 TBI) cohorts. In the EEG cohort, electrodes were implanted immediately after the induction of lateral FPI. Starting immediately after the TBI, video-EEG was performed first for 1 month and then during the 1st week of months 2 through 6. In the 7th post-TBI month, the rats underwent a 24 h/7 days video-EEG to record unprovoked spontaneous seizures (i.e., confirm epilepsy diagnosis). In the MRI cohort, the rats were imaged at 2, 9, and 30 days and at 5 months post-TBI; electrodes were implanted 14.02 ± 1.34 days (range: 4–40 days) after the last MRI, and video-EEG monitoring was performed during the 7th post-TBI month to detect unprovoked seizures (Figure 1A). Both groups were killed at the end of the 7-month follow-up, and the brains were processed for ex vivo MRI and histological analysis (Figure 1).

The EEG cohort was used to assess the sensitivity and specificity of ex vivo MRI to determine the electrode tip locations compared with histology. The MRI cohort was used to assess both (a) the accuracy of in vivo MRI-guided electrode implantation at the chronic phase and (b) the sensitivity and specificity of ex vivo MRI to detect electrode tip locations. Finally, we compared the accuracy of early (EEG cohort) and late (MRI cohort) electrode implantations.

### 2.2. Induction of Lateral FPI

Severe TBI was induced by lateral FPI as previously described [26,27]. The rat was placed into an anesthesia induction chamber and isoflurane anesthesia was induced at 5% (room air as carrier gas; Somnosuite, SS6069B, Kent Scientific, Torrington, CT, USA). The anesthetized rat was mounted in a stereotaxic frame, a probe was inserted into the rectum to continuously assess core temperature, and a heating pad was placed below the abdomen. The temperature of the heating pad was regulated based on the animal core temperature (max 38 °C). Isoflurane was delivered via a nose cone mounted on the stereotaxic frame and maintained at 1.9% throughout the surgery. The scalp incision site was shaved and cleaned using sterile 0.9% NaCl before subcutaneous injection with 0.5% lidocaine (7 mg/kg). Approximately 3 to 5 min later, a midline incision was made, and the surface of the skull was cleaned. A craniotomy 5 mm in diameter (center coordinate: anteroposterior [AP] −4.5 mm from the bregma; mediolateral [ML] 2.5 mm) was made over the left cortex using a handheld trephine with the dura left intact. A plastic female Luer lock connector was inserted into the craniotomy vertical to the skull surface, and its edges were sealed with tissue adhesive glue (3M Vetbond, 3M Deutschland GmbH, Neuss, Germany). Dental acrylate was spread around the Luer lock and the connector setup was secured to the skull with an ipsilateral frontal screw. To induce TBI, the Luer lock was filled with saline and the rat was connected to a straight-tipped fluid-percussion device (model FP 302, AmScien Instruments, Richmond, VA, USA). The pressure was adjusted to produce severe injury with an anticipated mortality rate of 20% to 30% within the first 48 h. The mean impact pressure was 2.9 ± 0.01 atm (range: 2.4–3.3 atm). The duration of the impact was <1 s. After injury, the rat was disconnected from the device, placed on a heating pad, and a rectal temperature probe was inserted. Occurrence of postimpact seizure-like behavior and its duration, duration of postimpact apnea, and time to return of the righting reflex were recorded. Sham-operated controls underwent all surgical procedures except exposure to FPI. To reduce impact of experimenter-induced variability in the experimental outcome, all surgical procedures, including TBI induction and electrode implantation, were performed by the same person.

### 2.3. Electrode Implantation

*EEG cohort.* Electrodes were implanted immediately after the lateral FPI using coordinates based on a rat brain atlas (Figure 1B,C) [25]. In brief, after return of the righting reflex, the rat was reanesthetized with isoflurane and placed in a stereotaxic frame. Four recording epidural stainless-steel screw electrodes (EM12/20/SPC; P1 Technologies, Roanoke, VA, USA) were implanted into the skull: 2 ipsilaterally (frontal cortex; C3, AP: −1.7, ML: left 2.5 and parieto-occipital cortex; O1, AP: −7.6, ML: left 2.5) and 2 contralaterally (frontal cortex; C4, AP: right 1.7; ML: −2.5 and parieto-occipital cortex; O2, AP: −7.6; ML: right 2.5; Figure 1B). Three intracerebral tungsten bipolar recording electrodes (EM12/3-2TW/SPC; P1 Technologies.; Ø 0.5 mm, tip separation 1.0 mm) were implanted ipsilaterally in the anterior perilesional cortex (AP: −1.72; ML: −4.0; DV: 1.8), septal hippocampus (AP: −3.0; ML: −1.4; DV: 3.6), and posterior perilesional cortex (AP: −7.56; ML: −4.0; DV: 1.8; Figure 1C). In addition, 1 epidural screw electrode serving as a ground was placed ipsilaterally posterior to lambda, and another serving as a reference electrode was placed contralaterally. Atlas-based placement of electrodes relies on calculating the target coordinates based on fixed skull surface landmarks, i.e., identification of bregma and midline sutural landmarks. Thus, the coordinates are defined independently of the intracranial changes in brain volume and orientation, which progress over time after TBI.

*MRI cohort.* Electrodes were implanted approximately 6 months (164 ± 1.4 days, range: 156–195 days) post-TBI. The locations of the epidural recording screws, ground, and reference electrodes were the same as in the EEG cohort described above. Due to progressive brain atrophy and ventricle enlargement, we first assessed the severity of cortical and hippocampal atrophy in each rat on MRI T2-weighted (T2-wt) images to define the AP, ML, and DV coordinates to avoid electrode misplacements (see below) (Figure 1D). Based on the analysis, the distance between the tips of the bipolar electrodes was reduced from 1.0 mm to 0.5 mm to fit within the atrophied cortex (upper tip in layer I, lower tip in layer V) or hippocampus (upper tip in CA1, lower tip in hilus).

### 2.4. Postoperative Care and Body Weight Monitoring

After surgery, rats were placed on a body-temperature–regulated heating pad (+38 °C) controlled by the SomnoSuite system (SS6069B, Kent Scientific). A subcutaneous injection of buprenorphine (0.01 mg/kg, Temgesic^®^, Reckitt&Colman Products Ltd., Hull, UK) and 0.9% NaCl (saline) was administered. Analgesia treatment was repeated based on animal well-being. Upon return to the home cage, rats received either powder or soft pellet food (until they could eat on their own) and saline (twice daily for 3 days). Rats were weighed daily for the first 14 days post-TBI, weekly until 30 days post-TBI, and then once a month throughout the 6-month follow-up. Adverse complications following TBI were monitored using a strict physiologic monitoring paradigm to assess general appearance; hair, coat and skin condition; bowel and gastrointestinal function; body conditioning score; condition of injury scar; and external bleeding. Any identified complication was treated according to the laboratory animal center’s guidelines.

### 2.5. Video-EEG Monitoring and Analysis

The video-EEG monitoring schedule is summarized in Figure 1. In the EEG cohort, monitoring was started immediately after surgery. In the MRI cohort, video-EEG monitoring was started at 6 months postinjury.

*Monitoring.* For video-EEG monitoring, the electrode headset attached to the rat skull was connected to a 12-pin swivel commutator (SL12C, PlasticsOne Inc., Roanoke, VA, USA) via a flexible shielded cable (363/2-363/2, PlasticsOne Inc.), allowing the rat to move freely during the EEG recordings. The commutator was connected to an amplifier with a flexible shielded cable 363/2-441/12 (PlasticsOne Inc). High-fidelity electrical brain activity was monitored using a 320-channel Digital Lynx 16SX amplifier (Neuralynx, Bozeman, MT, USA) with a 10 kHz sampling rate. The amplifier had an analogue bandwidth between DC to 80 kHz. It had 80 independent analogue references, allowing for a configuration of independent references for each animal. Data from each channel were converted individually into 24 bits. Each animal was video-monitored with a single high-resolution camera (Basler acA1300-75gm GigE, Basler, Germany) configured to record 30 frames per second (maximum 75) with a resolution of 1.3 megapixels and compressed using H.264. At night, cameras recorded under cage-specific infrared illumination (24 V, 150 mA). The EEG and video were synchronized using the precision time protocol IEEE-1588. The entire system generated approximately 1.5 TB of data every 24 h. For data storage, the video-EEG system was connected to network-attached storage (Synology RS4017xs+) comprising 200 TB of storage configured at RAID6 for redundancy and checksum for data integrity. The video-EEG recorded starting at 7 days after electrode implantation was analyzed for unprovoked seizures.

*Analysis of EEG.* Seizures were detected from video-EEG recordings by browsing the files visually and using the semiautomatic seizure detection algorithm [28]. A seizure was defined as a high-amplitude rhythmic discharge with frequency and amplitude modulation that clearly represented an abnormal EEG pattern (repetitive spikes, spike-and-wave discharges, polyspike-and-wave, or slow waves) and lasted at least 10 s. Behavioral severity of electrographic seizures was scored according to Racine [29]. Rats were defined as having epilepsy if at least 1 unprovoked electrographic seizure occurred during the 6-month EEG recording [30]. Seizure frequency was calculated as the number of unprovoked seizures/number of recording days.

### 2.6. MRI Acquisition

The animals were imaged in vivo using a 7-Tesla Bruker PharmaScan MRI scanner (Bruker BioSpin MRI GmbH, Ettlingen, Germany). The animals were anesthetized using 1% to 2% isoflurane mixed with carrier gas (70% N2, 30% O2), keeping their breathing rate at 50–70 breaths/min. The animal bed was heated to maintain the core temperature at 36–37 °C.

The MRI sequences used were described previously [31] [https://doi.org/10.1016/j.eplepsyres.2019.01.001, accessed 7 August 2022]. Briefly, T2-wt images were acquired using a 2D multislice fast-spin echo sequence with a repetition time of 3.4 s and an effective echo time of 45 ms. The image stack included 23 coronal slices with 800 µm thickness and an in-plane resolution of 117 µm × 117 µm. Additionally, 3D multigradient echo (MGRE) images with whole brain coverage at resolution 160 µm × 160 µm × 160 µm were acquired. Thirteen echoes with echo times between 2.7 ms and 40 ms were collected with a repetition time of 66 ms and a flip angle of 16°. The echo images were summed to create a high signal-to-noise ratio anatomic image.

### 2.7. MRI-Based Adjustment of Electrode Coordinates

As post-TBI cortical atrophy is common in the lateral FPI model, especially in the caudal aspects of the cortex [32], T2-wt MR images obtained at 5 months post-TBI were used to determine the anteroposterior (AP), mediolateral (ML), and dorsoventral (DV) coordinates of the depth electrodes in the MRI cohort. The aim was to position both tips of each bipolar depth electrode (0.5 mm vertical tip separation) in the rostral and caudal perilesional cortex or the septal hippocampus, instead of the lesion cavity or enlarged ventricles.

*Intracortical electrodes.* The 800 µm-thick in vivo T2-wt MRI coronal slices (11 slices from bregma to lambda) were converted to TIFF images. The images were uploaded into ImageJ software (version 1.47v, Wayne Rasband and contributors, National Institute of Health, USA), and scaled and aligned with the coronal plates of the rat brain atlas [25]. Then, the AP level (slice) near the planned anterior/posterior electrode location in which the cortex around the lesion cavity was thick enough to harbor both electrode tips (i.e., about 1 mm) was determined. The TIFF image (MR image) of that slice was used to define the ML and DV coordinates of the lower electrode tip using the ImageJ software line tool. First, a horizontal line was drawn from the pial surface of the brain midline laterally until its perpendicular vertical level reached layer V of the perilesional cortex approximately 500 µm from the lesion edge (Figure 1D). The horizontal length of the line was recorded as the ML coordinate. Then, the length of the vertical line to layer V was recorded as the DV coordinate (see details in [33]).

*Intrahippocampal electrodes.* An MRI T2-wt slice of the septal hippocampus approximately 3 to 4 mm from the bregma was selected. The MRI slice was opened in ImageJ as a TIFF file. To determine the ML coordinate, a horizontal line was drawn from the pial surface of the brain midline laterally until its perpendicular vertical level reached the hilus of the dentate gyrus. The horizontal length of the line was recorded as the ML coordinate (Figure 1D). The length of the vertical line to the hilus was recorded as the DV coordinate (see details in [33]).

The procedures were repeated for each rat because the lesion distribution as well as the cortical and hippocampal atrophy varied between rats.

### 2.8. MRI-Based Estimation of Cortical and Hippocampal Shrinkage

In the initial MRI-guided calculations of the AP and ML electrode coordinates, we did not consider tissue shrinkage or hippocampal transformation (rotation, tilting), which occurred over the follow-up and apparently contributed to some electrode misplacements. To improve the success rate, we next assessed the AP and ML shrinkage of both the ipsilateral and contralateral cortex and the hippocampi to further adjust the calculations for optimizing the electrode positions in the MRI cohort.

*AP shrinkage.* The coronal, sagittal, and horizontal in vivo MR 3D MGRE slices (160 µm thick) of each animal in the MRI cohort were opened in the medical images analysis application Aedes (version 1.0 rev 218, GitHub: aedes_getfilefilter.m, Juha-Pekka Niskanen, University of Eastern Finland, Finland), using MATLAB software (version R2019b, The MathWork, Inc, Natick, MA, USA). In the sagittal slices 4 mm from the midline (i.e., ML coordinate of the anterior intracortical electrode), the AP length of the ipsilateral and contralateral cortex was measured by calculating the geometric distance between the 2 points (160 × 160 × 160 µm resolution images) in Aedes (see detail in Section 3.4.1)

*ML shrinkage.* To set the ML level for the measurement, the targeted AP location of the rostral intracortical electrode was used as a reference (AP −1.72). Typically, it was located in a slice that was 1.56 mm (i.e., approximately ten 160 µm-thick MRI slices) caudal to the level where the ipsilateral and contralateral legs of the anterior commissure fuse, which occurs approximately at the level of the bregma (see detail in Section 3.4.1). Then, the ML distance from the midline to the lateral edge of the cortex was measured in a horizontal slice 1.7 mm below the surface of the brain (targeted DV location of the lower electrode tip; see detail in Section 3.4.1).

*Hippocampal shrinkage.* The AP hippocampal distortion was measured in the sagittal slice 1.4 mm from the midline as the distance from the rostral edge of the frontal cortex to the rostral edge of the hippocampus (see detail in Section 3.4.2) The ML shrinkage was measured as the distance from the brain midline to the lateral edge of the hippocampus in a horizontal slice at 2.8 mm below the brain surface (see details in Section 3.4.2).

### 2.9. Histology

At the end of the 7-month follow-up period, all rats were transcardially perfused with saline (5 min, 30 mL/min) followed by cold 4% paraformaldehyde (PFA; 30 min, 30 mL/min). The brain was removed from the skull, postfixed in 4% PFA (2 h at 4 °C), and cryoprotected by immersing in 20% glycerol for 36 h (4 °C). The brains were then frozen on dry ice and stored at −70 °C until further processing. The brains were sectioned at 30 µm in a 1-in-5 series using a sliding microtome.

The first series of sections was stained with thionine, mounted on glass slides, and cover-slipped from xylene. The mounted thionine-stained sections were scanned at 40× magnification with a digital slide scanner (Hamamatsu C12000-02 model) and analyzed using the Hamamatsu NDP viewer 2^®^ software (Hamamatsu Photonics K.K., Hamamatsu City, Japan).

### 2.10. Generation of Unfolded Cortical Maps

The unfolded cortical maps were generated from coronal histological sections using software developed in-house, as described previously [32,34,35]. The unfolded maps were used to determine (a) the location of the cortical lesion in various cytoarchitectonic fields of the cortical mantle, (b) the total lesion area, (c) the lesion area within different cortical cytoarchitectonic subareas, (d) the planned vs. final AP and DV coordinates of the intracerebral electrodes, and (e) the distance of the electrode tips from the lesion edge. The unfolded map software is openly available at https://www.unfoldedmap.org/ (accessed 30 November 2020) [34] and the source code at https://github.com/UEFepilepsyAIVI/CortexMap (accessed 30 November 2020) [35].

*Histological sections.* To determine the final AP and DV locations of the electrode tip(s), the digitized image of the histological section(s) containing a trace of the electrode tip was matched with the best-fitting level of the rat brain atlas.

As the ML coordinate cannot be easily determined based on the atlas due to shrinkage related to histological processing, we first estimated the shrinkage factor for each animal by measuring the distance from the midline to the rhinal fissure in the histological section containing the electrode tip. The midline-hinal fissure distance was then measured at the matching atlas plate (EEG cohort) or T2-wt MRI slice (MRI cohort) and divided by the corresponding histological measure, resulting in a shrinkage factor specific for each brain (sham: 1.08 ± 0.01, range: 1.02–1.2, *n* = 25; TBI: 1.08 ± 0.01, range: 1.01–1.2, *n* = 74). Cortical and hippocampal electrode tip distances from the midline were then measured in histological sections and multiplied by the shrinkage factor to obtain the normalized ML distance.

To measure the distance of the electrode tip from the lesion cavity edge, a straight line was drawn from the tip to the lesion edge along layer V.

### 2.11. Statistical Analysis

Data were analyzed using GraphPad Prism (version 9.3.1, GraphPad Software, LLC, USA) and IBM SPSS Statistics (version 27, IBM Corp., USA). A Shapiro–Wilk test was performed to test for normality. The data were not normally distributed. Thus, the Mann–Whitney *U* test was used to analyze (1) the difference between the atlas, histological, and/or MRI-based coordinates (sham vs. TBI rats, EEG and MRI cohorts), (2) the difference in the distance of the electrode tip from the lesion edge (TBI rats, EEG vs. MRI cohorts), and (3) the difference in cortical and hippocampal shrinkage (sham vs. TBI rats, MRI cohort). The Wilcoxon signed-rank test was used to test the difference between the targeted and “true” histologically verified tip coordinates between the anterior and posterior cortical electrodes ipsilateral vs. contralateral cortical and hippocampal shrinkage. The Pearson chi-squared statistic was used to test the difference in the distribution of the DV location (layers) of the lower tip of the intracortical electrodes (sham vs. TBI rats, anterior vs. posterior cortical electrodes, separately in the EEG and MRI cohorts). All data are presented as means ± SEM.

## 3. Results

Altogether, the analysis included the location of 99 anterior intracortical, 99 posterior intracortical, and 99 intrahippocampal electrodes implanted in 99 rats (57 in the EEG cohort and 42 in the MRI cohort). In the EEG cohort, electrodes were implanted immediately after the lateral FPI using coordinates based on a rat brain atlas (Figure 1A–C). In the MRI cohort, electrodes were implanted approximately 6 months post-TBI using MRI T2-weighted (T2-wt) images from each rat to define the AP, ML, and DV coordinates to avoid electrode misplacements (see below) (Figure 1A,B,D).

### 3.1. Success in Positioning the Anterior Intracortical Electrode

#### 3.1.1. Electrode Locations—An Overview

**Figure 2:**
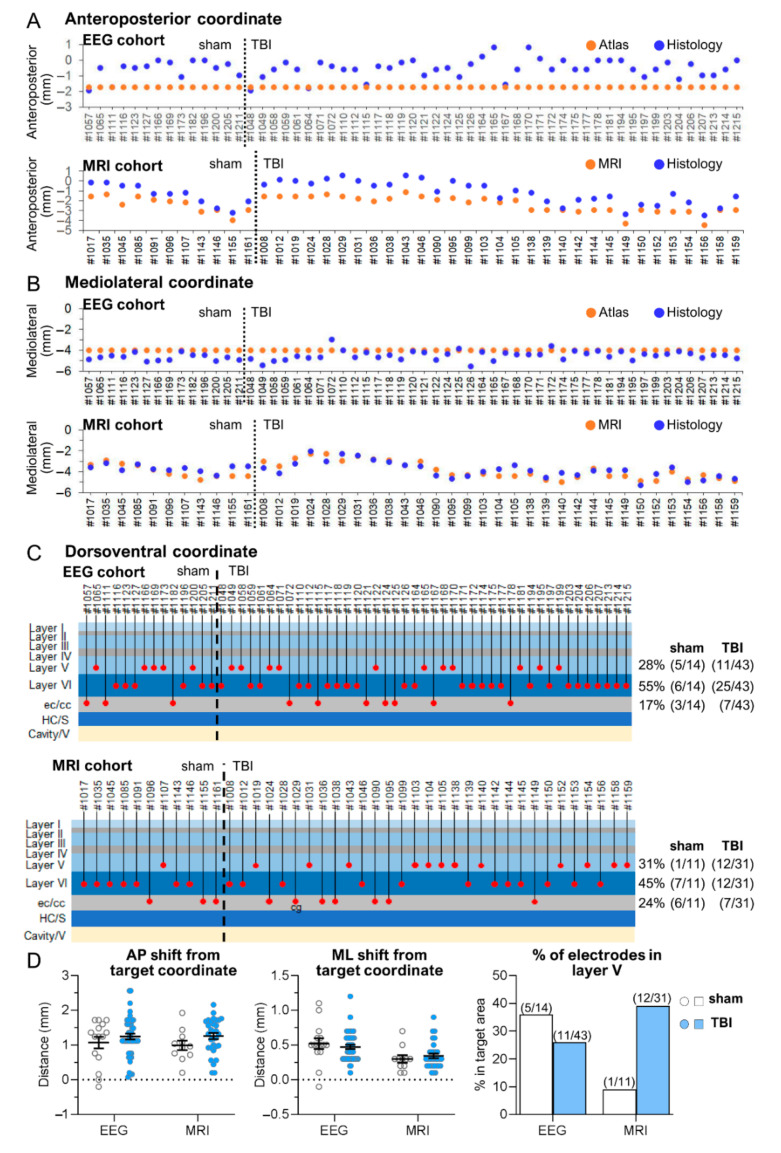
Anterior intracortical electrode—schematic representations of the atlas, histological, and MRI-guided coordinates in each rat of the EEG and MRI cohorts. (**A**) Anteroposterior (AP) coordinate. In the EEG cohort (electrode operation right after injury), the fixed atlas-based target AP coordinate of −1.72 mm from the bregma was applied to implant the electrodes (orange dots). In the MRI cohort (electrode operation 5 months after injury), the target AP coordinate was individually determined using the 5-month in vivo T2-weighted MR images. The target coordinate fluctuated depending on the extent of the TBI (traumatic brain injury)-induced lesion. Note the anterior shift (*y*-axis) in the histologically verified “true” AP coordinate (blue dots) relative to the target coordinate (orange dots) in both cohorts. The deviations were comparable between the sham and TBI animals (*p* > 0.05). Animal numbers are shown on the *x*-axis. (**B**) Mediolateral (ML) coordinate. In the EEG cohort, the fixed atlas-based target ML coordinate at 4 mm lateral to midline was targeted. In the MRI cohort, the target ML coordinate was individually determined using the 5-month in vivo MRI. Note a small deviation of the histologically verified “true” ML coordinate from the target coordinate in both cohorts. (**C**) Dorsoventral (DV) coordinate. In both cohorts, the lower tip of the bipolar electrode was aimed to layer V in the selected AP and ML coordinates (see above). Electrode tips were located in the cortex in 83% (36/43) of the EEG cohort and 77% (24/31) of the MRI cohort. Importantly, even though the lower tip in the remaining cases went down into the external capsule or corpus callosum, the upper tip of the bipolar electrode, being 1 mm higher in the EEG and 0.5 mm in the MRI cohort, was still recording in the cortex. The percentages of electrode locations in the sham-operated and TBI animals are shown on the right side of the panel. (**D**) Dot plots of the AP and ML shift in the histological AP and ML coordinate, and % of electrode in the targeted layer V (number of cases in brackets). Note posterior and medial shift of some cases from the target (vertical dashed line). *y*-axis represents distance from target coordinate (Y = 0) or % of cases in targeted area. Abbreviations: cavity, cortical lesion cavity; cc, corpus callosum; cg, cingulum; S, subiculum; ec, external capsule; HC, hippocampus; and V, ventricle.

*EEG cohort.* Histological analysis indicated that the tip of the anterior intracortical electrode was within the cerebral cortex in 83% (47/57) of the cases. In the remaining 17% (10/57), the tip was located in either the external capsule or the corpus callosum. The histologically verified AP and ML electrode tip locations were within 0.5 mm of the targeted atlas-based coordinates in 11% (6/57) and 65% (37/57) of the rats, respectively (Figure 2A,B). The histological DV location of the cortical electrode tip was in the planned target (i.e., layer V) in 28% (16/57) of the animals. In the remaining 72% (41/57), the tip was located in either layer VI, the external capsule, or the corpus callosum (Figure 2C and summary Table 1).

*MRI cohort.* Histological analysis indicated that the tip of the anterior intracortical electrode was within the cerebral cortex in 76% (32/42) of the cases. In the remaining 24% (10/42), the tip was located in either the external capsule or the corpus callosum. The histological AP and ML electrode tip locations were within 0.5 mm of the targeted atlas-based coordinates in 10% (4/42) and 86% (36/42) of the rats, respectively (Figure 2A,B). The histological DV location of the cortical electrode tip was in the planned target (i.e., layer V) in 31% (13/42) of the animals. In the remaining 69% (29/42), the tip was located in either layer VI, the external capsule, or the corpus callosum (Figure 2C and summary Table 1).

#### 3.1.2. Anteroposterior Location of the Anterior Intracortical Electrode Tips

Based on estimation of the progression of the cortical lesion [7,27,32], the anterior intracortical electrode was aimed to the atlas-based AP level 1.72 mm from the bregma (primary somatosensory cortex, S1) to ensure proper EEG recording in the lesion vicinity during the 7th post-TBI month (Figure 1C).

**Figure 3:**
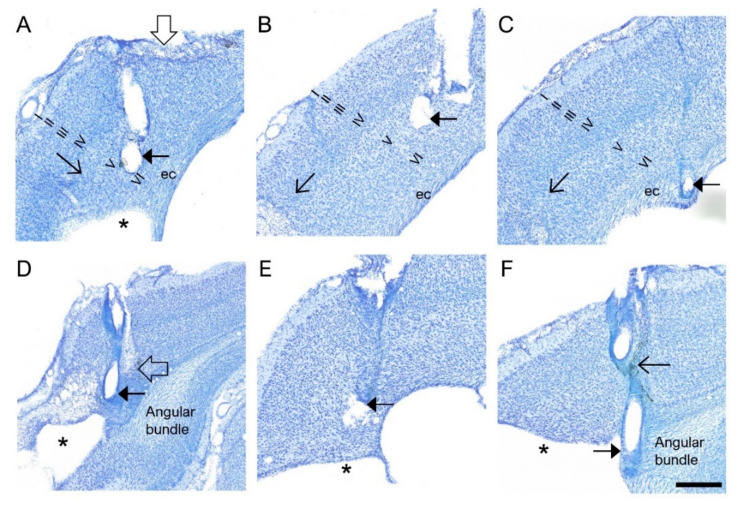
(**A**–**F**) Electrode tracts. Histological images from the coronal thionine-stained sections of 6 rats, showing the tracts of the bipolar intracortical electrodes and location of the lower electrode tip (filled arrowhead). Roman numerals indicate the cortical layers. In panels (**A**,**B**) the electrode tip is located in layer V of the perilesional cortex. Note the electrode track-related lesion on the surface of the brain in panel (**A**) (open filled arrow). In panel (**A**), the electrode tip is within 500 µm from the edge of the TBI-induced lesion cavity (asterisk). In panel (**C**), the electrode tip is in the external capsule (ec). In panel (**D**) the electrode tip is within the cortical lesion, close to the angular bundle. Open arrow points to the electrode path associated neurodegeneration. In panel (**E**), the electrode tip is close to the edge of the lesion cavity (asterisk). In panel (**F**), the electrode tip is within the angular bundle (closed arrowhead). The open arrowhead points to the location of the upper electrode in layer IV (open arrow). The dark staining indicates iron deposits (arrowheads) adjacent to the electrode path. Scale bar = 500 µm.

**Figure 4:**
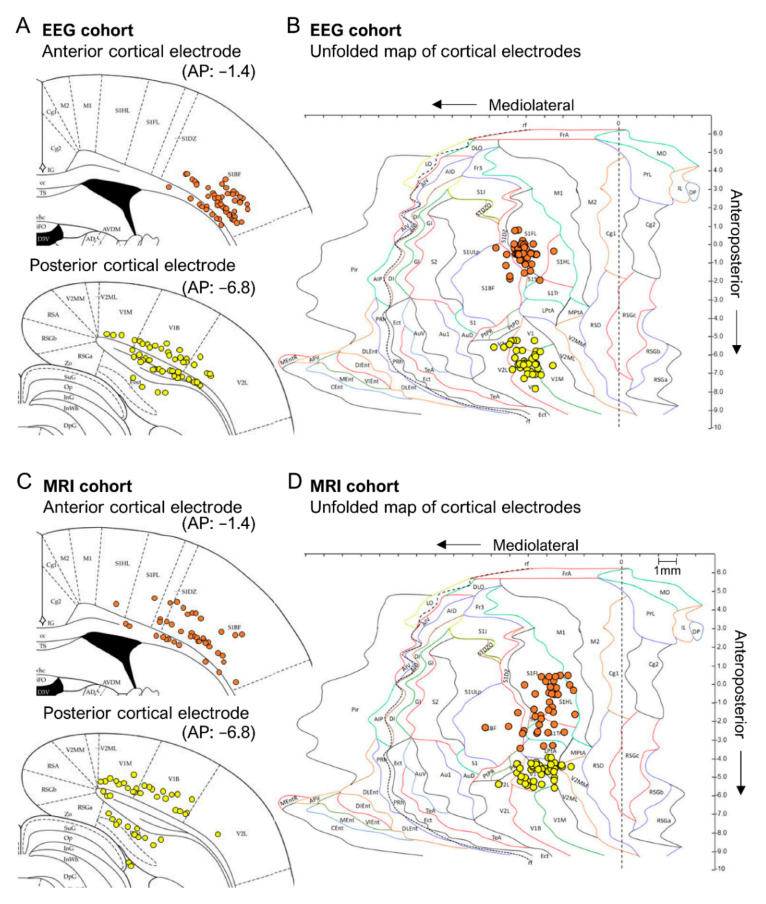
Location of the lower tip of the anterior and posterior intracortical electrodes on atlas plates and unfolded cortical maps. (**A**) In the EEG cohort (upper panel), the dorsoventral (DV) location of at least 1 of the tips of all anterior bipolar electrodes (atlas plate: bregma −1.4 mm) was within the primary somatosensory cortex (S1) and that of the posterior electrode (lower panel; atlas plate: bregma −6.8 mm) was within the visual cortex. Each dot represents 1 bipolar electrode. (**B**) An unfolded map (UFM) showing the location of electrode tracks in the EEG cohort as seen from the surface of the brain. The intersection of the electrode path with cortical layer V was used as reference. The UFMs confirmed the location of the anterior electrode paths in the S1 and posterior electrode paths in the visual cortex. (**C**) Atlas plate showing the DV locations of the anterior (upper panel) and posterior (lower panel) intracortical electrodes in the MRI cohort. As in the EEG cohort, the anterior electrode was in S1 and the posterior electrode was in the visual cortex. (**D**) A UFM showing the location of electrode tracks in the MRI cohort as seen from the surface of the brain. All electrode tracks were within S1 or the visual cortex. Note that in the MRI cohort, we used the 5-month in vivo MRI to adjust the electrode coordinates to target the perilesional cortex and to avoid lesion cavities, underlying brain areas, or ventricles. As expected, this resulted in a more heterogeneous distribution of electrode paths than in the EEG cohort with atlas-based fixed coordinates. Atlas plates and UFMs were generated using the Paxinos rat brain atlas (6th edition).

*EEG cohort.* Histological examination showed neurodegeneration along the electrode path and around the electrode tip, often accompanied by iron deposits (Figure 3A,D,F). Occasionally, the electrode-associated lesion merged with the TBI-induced lesion cavity. The unfolded cortical map indicated that 100% of the electrode tracks were in S1. Further, 83% (47/57) of the electrode tips (at least one of the tips) were found in S1 (Figure 4A,B). The remaining 17% (10/57) of the lower electrode tips were located in either the external capsule or the corpus callosum (Figure 2C and Figure 4A,B). The upper tip of the bipolar electrode (1.0 mm from the ventral tip), however, recorded in the targeted S1 cortex. In most of the rats, the histological AP electrode location was anterior to the targeted atlas-defined coordinate (−1.7 mm) (Figure 2A,D), the average deviation being 1.21 ± 0.07 mm (range: 0–2.56 mm, median: 1.24 mm). The rostral shift was comparable between the sham-operated experimental controls and TBI rats (sham 1.16 ± 0.15 mm vs. TBI 1.22 ± 0.09 mm; *p* > 0.05) (Figure 2A,D).

*MRI cohort.* Electrode-associated cortical lesions were rare in the MRI cohort (Figure 3A,D). As summarized in the cortical unfolded map, 100% of the electrode tracks (100%) and 76% (32/42) of the electrode tips were within S1 (Figure 4C,D). In the remaining cases, the lower tip was located within the external capsule or the corpus callosum (Figure 4C,D). Still, the upper tip of the bipolar electrode (0.5 mm from ventral tip) should have recorded in the targeted S1 cortex. As in the EEG cohort, the histological electrode location was anterior to the targeted MRI-guided AP coordinate, the average being 1.18 ± 0.08 mm (range: 0.2–2.16 mm, median: 1.19 mm) (Figure 2A,D). The rostral shift was comparable between the sham-operated controls and TBI groups (sham 0.99 ± 0.14 mm vs. TBI 1.25 ± 0.09 mm; *p* > 0.05) (Figure 2A,D).

#### 3.1.3. Mediolateral Location of the Anterior Intracortical Electrode Tips

*EEG cohort.* Histological assessment revealed that the tip was lateral in 93% (53/57) of the cases and medial to the atlas-based coordinates (4 mm from midline) in 7% (4/57) of the cases (Figure 2B). The mean distance between the histological and MRI-guided ML coordinate was 0.58 ± 0.05 mm (range: 0–1.50 mm, median: 0.5 mm) (Figure 2D). The distance was comparable between sham-operated controls (0.68 ± 0.09 mm) and TBI (0.55 ± 0.06 mm) rats (*p* > 0.05) (Figure 2D).

*MRI cohort.* Histological assessment revealed that the tip was lateral to the MRI-based coordinates in 41% (17/42) of the cases and medial in 59% (25/42) of the cases (Figure 2B). The mean distance between the histological and MRI-guided ML coordinate was 0.44 ± 0.04 mm (range: 0–1.0 mm, median: 0.4 mm) (Figure 2D). The distance was comparable between the sham-operated controls (0.48 ± 0.09 mm) and TBI rats (0.42 ± 0.05 mm; *p* > 0.05) (Figure 2D).

#### 3.1.4. Dorsoventral Location of the Anterior Intracortical Electrode Tips

Cortical layer V was set as the DV target for the lower tip of the bipolar intracerebral electrode in both cohorts.

*EEG cohort.* Histological analysis revealed that 83% (47/57) of the electrodes had at least one tip within the cerebral cortex. Of the 47 electrodes, 28% (16/57) were in layer V and 55% (31/57) were in layer VI. In the remaining 17% (10/57), the tip was identified in either the external capsule or the corpus callosum (Figure 2C). The DV distribution of the electrode tips was comparable between sham and TBI rats (ꭕ^2^ test; *p* > 0.05) (Supplementary Table S1).

*MRI cohort.* The DV location of the electrode tip was within the cortex in 76% (32/42) of rats, being in layer V in 31% (13/42) and layer VI in 45% (19/42) of the cases. In the remaining 24%, the tip was located in either the corpus callosum (7%, 3/42) or the external capsule (17%, 7/42) (Figure 2C). The DV distribution of the electrode tips was comparable between sham and TBI rats (ꭕ^2^ test; *p* > 0.05).

### 3.2. Success in Positioning the Posterior Intracortical Electrode

#### 3.2.1. Electrode Locations—An Overview

**Figure 5:**
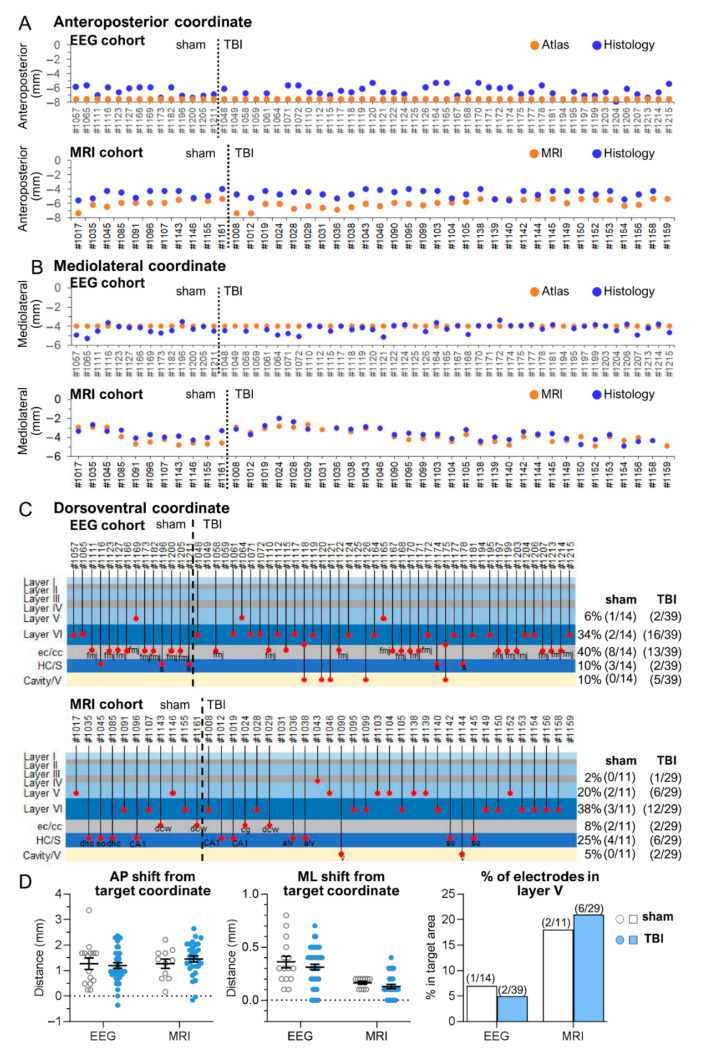
Posterior intracortical electrode—schematic representations of the atlas-based, histological, and MRI-guided coordinates in each rat of the EEG and MRI cohorts. (**A**) Anteroposterior (AP) coordinate. In the EEG cohort (*n* = 47, electrode operation right after injury), the fixed atlas-based target AP coordinate of −7.56 mm from the bregma was applied to implant the electrodes (orange dots). In the MRI cohort (*n* = 40, electrode operation at 5 months postinjury), the target AP coordinate was individually determined using the in vivo 5-month T2-weighted MR images. The target coordinate fluctuated depending on the TBI (traumatic brain injury)-induced lesion extent. Note a mild anterior shift (*y*-axis) in the histologically verified “true” AP coordinate (blue dots) relative to the target coordinate (orange dots) in both cohorts. In general, the anterior shift was less than that in a case of the anterior intra-cortical electrode (compare to Figure 1). Animal numbers are shown on the *x*-axis. (**B**) Mediolateral (ML) coordinate. In the EEG cohort (*n* = 47), the fixed atlas-based ML coordinate at 4 mm lateral to midline was targeted. In the MRI cohort (*n* = 40), the target ML coordinate was individually determined using the 5-month MRI. Note almost a negligible deviation of the histologically verified “true” ML coordinate from the atlas-based (EEG cohort) or MRI-guided (MRI cohort) coordinates. (**C**) Dorsoventral (DV) coordinate. In both cohorts, the lower tip of the bipolar electrode was targeted to layer V in the selected AP and ML coordinates (see above). In the EEG cohort, 46% (18/39), and in the MRI cohort, 66% (19/29) of the electrode tips in injured animals were in the cortex. Importantly, even though the lower tip in the remaining cases went down into the external capsule or corpus callosum, the upper tip of the bipolar electrode, being 1 mm higher in the EEG and 0.5 mm in the MRI cohort, was still recording in the cortex in 79% (31/39) of the rats in the EEG cohort and in 72% (21/29) in the MRI cohort. In 5 rats (3 sham, 2 TBI) in the EEG cohort and 10 rats (4 sham, 6 TBI) in the MRI cohort, the electrode was recording hippocampal rather than cortical activity, which affected the interpretation of the EEG data. The percentages of electrode locations in the sham-operated and TBI animals are shown on the right side of the panel. (**D**) Dot plots of the AP and ML shift in the histological AP and ML coordinate, and % of electrode in the targeted layer V (number of cases in brackets). Note posterior shift of some TBI cases from the target (Y = 0). The *y*-axis represents distance from target coordinate (Y = 0) or % of cases in targeted area. Note that in 4 animals in the EEG cohort and 2 in the MRI cohort, the DV location of the electrode tip could not be reliably determined in histological sections. Abbreviations: cavity, cortical lesion cavity; cc, corpus callosum; cg, cingulum; dcw, deep cerebral white matter; ec, external capsule; fmj, forceps major corpus callosum; HC, hippocampus; S, subiculum; V, ventricle.

*EEG cohort.* The electrode tip was within the cortex in 40% (21/53) of the rats. In the remaining 60% (31/53), it was either in the angular bundle, dorsal subiculum, or cortical lesion cavity (Figure 5 and Table 1). In four rats, the quality of the histological sections was not sufficient to determine the electrode location. The histological AP tip and ML electrode tip locations were within a 0.5-mm radius of the planned atlas coordinates in 19% (10/53) and 89% (47/53) of the rats, respectively (Figure 5A,B). The DV tip location was in the planned depth (layer V) in only 6% (3/53) of the rats. In the remaining 94% (50/53), it was either in layer VI, the angular bundle, dorsal subiculum, or cortical lesion cavity (Figure 5C).

*MRI cohort.* The electrode tip was within the cortex in 60% (24/40) of the rats. In the remaining 40% (16/40), it was either in the external capsule, corpus callosum, dorsal subiculum, hippocampus, or ventricle. In two rats, the quality of the histological sections was not sufficient to determine the electrode location. The histological AP and ML electrode tip locations were within a 0.5 mm radius of the planned MRI coordinates in 8% (3/40) and 100% (40/40) of the rats, respectively (Figure 5A,B and Table 1). The DV tip location was in the planned depth (layer V) in only 20% (8/40) rats. In the remaining 80% (32/40), the tip was located in either layer IV or VI, the external capsule, corpus callosum, dorsal subiculum, hippocampus, or ventricle (Figure 5C).

#### 3.2.2. Anteroposterior Location of the Posterior Intracortical Electrode Tips

Based on estimation of the progression of the cortical lesion, which tends to be more extensive caudally than rostrally [7,27,32], the posterior intracortical electrode was targeted to the atlas-based AP level −7.56 from the bregma (primary visual cortex, V1) to ensure proper EEG recording in the lesion vicinity during the 7th post-TBI month (Figure 1A).

*EEG cohort.* The unfolded cortical map indicated that all electrode tracks passed through the visual cortex. The electrode tips were in the visual cortex in only 40% (21/53) of the rats. In the remaining 60% (31/53), they were in the angular bundle, dorsal subiculum, or cortical lesion cavity (Figure 4A,B and Figure 5C). Typically, the tip location was anterior to the targeted atlas-based AP coordinate, the average deviation being 1.16 ± 0.09 mm (range: 0–2.28 mm, median: 0.96 mm) (Figure 5A,D). The rostral shift was comparable between the sham-operated controls and TBI rats (1.11 ± 0.17 mm vs. 1.18 ± 0.10 mm; *p* > 0.05) (V). The rostral shift of the posterior intracortical electrode was comparable to that of the anterior intracortical electrode (*p* > 0.05).

**Figure 6:**
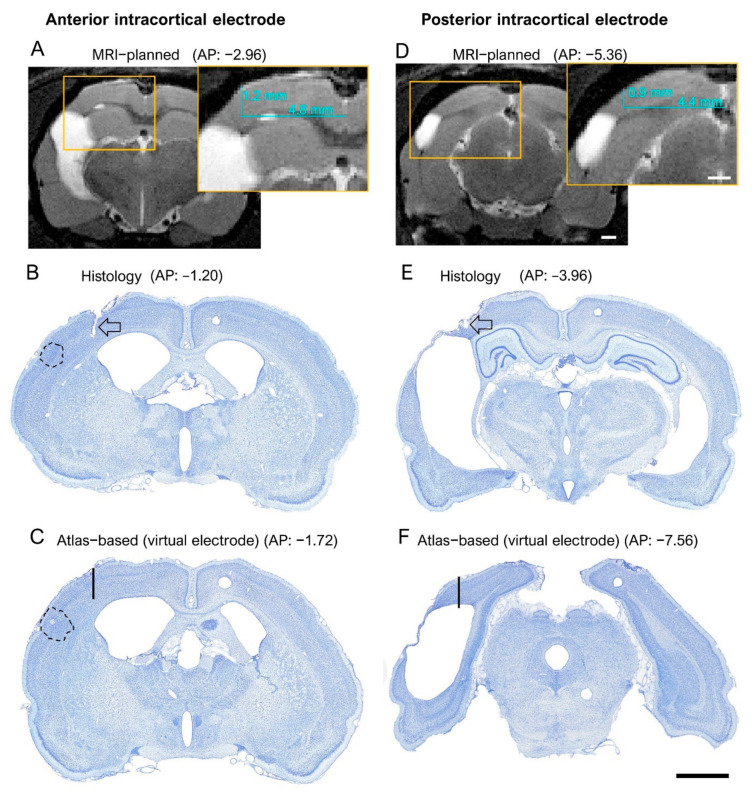
Histological confirmation of the success of MRI-guided electrode placement. Left panel (**A**–**C**): Anterior intracortical electrode MRI-planned coordinate, histological confirmation, and “virtual” electrode. Right panel: Posterior intracortical electrode MRI-panned coordinate histological confirmation, and “virtual” electrode. (**A**) T2-weighted MRI and (**B**) histological images showing the MRI-guided (insert) and histology-confirmed “true” location of the anterior intracortical electrode in rat 1139. The anteroposterior (AP) coordinate was estimated by aligning the magnetic resonance images with the rat brain atlas [25]. The mediolateral (ML) and dorsoventral (DV) coordinates (inserts in (**A**,**D**)) were determined using ImageJ software (version 1.47v, Wayne Rasband and contributors, National Institute of Health, USA). Note that in this case, the confirmed AP location was about 1.8 mm more rostral than the planned location (−1.20 mm vs. −2.96). Lesion area is denoted in black-dashed-line circle. (**C**) A “virtual” location of the electrode tip (black line) if the electrode had been implanted to the targeted atlas-based coordinate (−1.75 mm from bregma, 4 mm from midline, 1.8 mm from the surface of the brain). (**D**) MRI-guided (insert) AP, ML and DV coordinates and (**E**) histology-confirmed “true” location of the posterior intracortical electrode tip in rat 1139. The black-dashed-line circle denotes the lesion area. Note that the confirmed AP location was approximately 1.4 mm more rostral than the planned location (−3.96 vs. −5.36). Thus, even though both the anterior and posterior intracortical electrodes were more rostral than planned, their tips were recording EEG signals in the perilesional cortex. (**F**) A “virtual” electrode (black line) at the atlas-based coordinates would have ended up in the lesion cavity. Scale bar in (**A**,**D**) = 1 mm, and in (**B**,**C**,**E**,**F**) = 2 mm.

*MRI cohort.* As in the EEG cohort, histological analysis revealed that most of the posterior intracortical electrodes (60%, 24/40) were in the visual cortex. In the remaining 40% (16/40) of rats, the electrode track was in the visual cortex, but the tip penetrated either the deep cerebral white matter, cingulum, dorsal hippocampal commissure, hippocampus proper, or ventricle (Figure 4C,D and Figure 5C). In 95% (38/40) of the rats, the electrode tip was anterior to the targeted MRI-guided AP coordinate (Figure 5A,D and Figure 6A,B,D,E), the average deviation being 1.39 ± 0.09 mm (range: 1.28–2.64 mm, median: 1.42 mm). The rostral shift was comparable between the sham-operated controls and TBI rats (sham 1.26 ± 0.18 vs. TBI 1.45 ± 0.12; *p* > 0.05) (Figure 5D). The rostral shift of the posterior intracortical electrode was comparable to that of the anterior intracortical electrode (*p* > 0.05).

#### 3.2.3. Mediolateral Location of the Posterior Intracortical Electrode Tips

*EEG cohort.* The histological ML tip coordinate was lateral to the targeted atlas-based coordinate in 62% (33/53), on target in 2% (1/83), and medial in 36% (19/53) of the rats (Figure 4B). The mean deviation from the atlas-based ML coordinate (0.38 ± 0.05 mm, range: 0–1.26 mm, median: 0.3 mm) was less than that of the anterior intracortical electrode (0.58 ± 0.05 mm, range: 0–1.50 mm; *p* < 0.05) (Figure 5D). The average deviation was comparable between sham-operated controls (0.45 ± 0.09 mm) and TBI rats (0.35 ± 0.05 mm; *p* > 0.05) (Figure 5D).

*MRI cohort.* The histological ML tip coordinate was lateral to the MRI-guided coordinate in 25% (10/40) and medial in 75% (30/40) of the rats (Figure 4B). The mean distance between the histological ML and MRI-guided coordinate was 0.45 ± 0.04 mm (range: 0.03–1.29 mm, median: 0.4 mm). The deviation was comparable between sham-operated controls (0.62 ± 0.09 mm) and TBI rats (0.39 ± 0.04 mm; *p* > 0.05) (Figure 5D). Also, the deviation from the MRI-based ML did not differ from that of the anterior intracortical electrode (0.44 ± 0.04 mm; *p* > 0.05) (Figure 5D).

#### 3.2.4. Dorsoventral Location of the Posterior Intracortical Electrode Tips

*EEG cohort.* The histologically verified DV location of the lower tip of the posterior intracortical electrode was more diverse compared with the anterior electrode, with only 40% (21/52) within the cortical layers (6% in layer V, 34% in layer VI) (Figure 5C and Table 1). Moreover, 40% (21/52) of the tips had traveled down to the angular bundle, 10% (5/53) to the dorsal subiculum, and 10% (5/52) to the underlying cortical lesion cavity (Figure 5C).

Further analysis indicated that the laminar location of the electrode tips differed between the posterior and anterior electrodes (ꭕ^2^ test; *p* < 0.001). The overall percentage of posterior electrode tips in the cortex was less than that of the anterior electrode (40% vs. 83%; *p* < 0.001) (Table 1). Also, fewer posterior electrode tips were located in the targeted layer V compared with anterior cortical electrode tips (9% vs. 28%; *p* < 0.01) (Figure 5C, Supplementary Table S1). Surprisingly, the overall percentage of posterior cortical electrodes in the cortex was comparable between sham (46%; 5/11) and TBI rats (67%, 19/29; *p* > 0.05). Also, there was no difference in the overall DV distribution of the locations of the posterior intracortical electrode tips between sham and TBI rats (ꭕ^2^ test; *p* > 0.05) (Supplementary Table S1).

*MRI cohort.* Altogether, 60% (24/40) of the posterior intracortical electrodes had the lower tip within the cortex, of which 2% (1/40) were in layer IV, 20% (8/40) in layer V, and 38% (15/40) in layer VI (Figure 5C and Table 1). The tip had reached the external capsule or cingulum in 8% (4/40), the angular bundle or hippocampus proper in 27% (11/40), and the ventricle in 5% (2/40). Unlike in the EEG cohort, none of the electrode tips appeared to enter the lesion cavity (Figure 5C). The overall DV tip distributions did not differ between sham-operated controls and TBI rats (ꭕ^2^ test; *p* > 0.05) (Supplementary Table S1).

The overall percentage of tips in the cortex was comparable between the anterior and posterior cortical electrodes (76% vs. 60%, ꭕ^2^ test; *p* > 0.05), and between sham and TBI rats (46% vs. 66%, ꭕ^2^ test; *p* > 0.05) (Table 1). Like in the EEG cohort, however, the overall DV distribution of the electrode tips differed between the anterior and posterior intracortical electrodes (ꭕ^2^ test; *p* < 0.01). Particularly, the percentage of electrodes entering into the septal hippocampus was greater in the TBI rats than in the sham group (27% vs. 0%, ꭕ^2^ test; *p* < 0.001).

### 3.3. Success in Positioning the Intrahippocampal Electrode

#### 3.3.1. Electrode Locations—An Overview

The lower tip of the hippocampal electrode was targeted to the hilus of the septal end of the dentate gyrus (AP: −3.0; ML: −1.4; DV: 3.6) in both the EEG and MRI cohorts.

**Figure 7:**
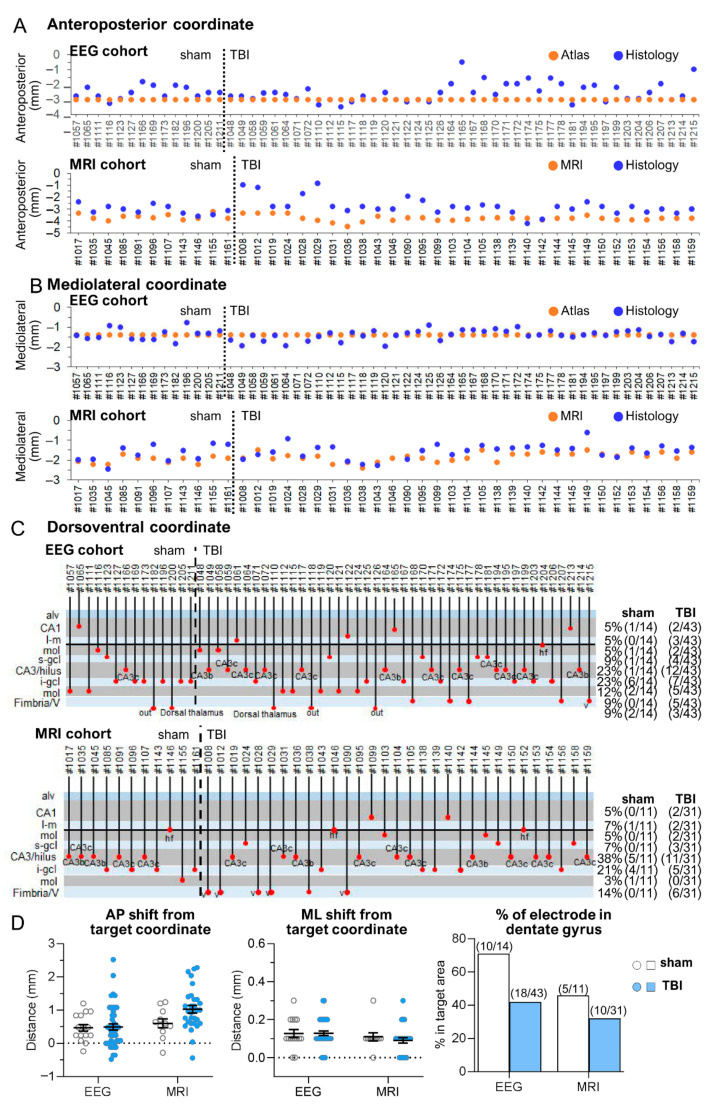
Hippocampal electrode—schematic representations of the atlas-based, histological, and MRI-guided coordinates in each rat of the EEG and MRI cohorts. (**A**) Anteroposterior (AP) coordinate. In the EEG cohort (electrode operation right after injury), the fixed atlas-based target AP coordinate of −3 mm from the bregma was applied to implant the electrodes (orange dots). In the MRI cohort (electrode operation 5 months after injury), the target AP coordinate was individually determined using the 5-month in vivo T2-weighted MR images. The target coordinate fluctuated, depending on the TBI-induced hippocampal structural abnormality. Note the anterior shift (*y*-axis) in the histologically verified “true” AP coordinate (blue dots) relative to the aimed target coordinate (orange dots) in both cohorts. Note the great variability in the anterior shift from animal to animal, particularly in the MRI cohort. Animal numbers are shown on the *x*-axis. (**B**) Mediolateral (ML) coordinate. In the EEG cohort, the fixed atlas-based target ML coordinate at 1.4 mm lateral to midline was targeted. In the MRI cohort, the target ML coordinate was individually determined using the 5-month MRI. Note only a very small deviation of the histologically defined “true” ML coordinate from the atlas-based (EEG cohort) or MRI-guided (MRI cohort) coordinates. (**C**) Dorsoventral (DV) coordinate. In both cohorts, the lower tip of the bipolar electrode was aimed at the hilus in the selected AP and ML coordinates (see above). In the EEG cohort, most of the tips were recording in the hippocampus proper or the dentate gyrus. In the EEG cohort, in only 19% (8/43) of TBI cases, the tip was either in fimbria, ventricle, or went through the septal hippocampus to the dorsal thalamus or to an unidentified location. In the MRI cohort, in only 14% (6/31) of TBI cases, the tip was outside the hippocampus or the dentate gyrus. The percentages of electrode locations in the sham-operated and TBI animals are shown on the right side of the panel. (**D**) Dot plots of the AP and ML shift in the histological AP and ML coordinate, and % of electrodes in the targeted dentate gyrus (number of cases in brackets). Note posterior shift of some cases from the target (vertical dashed line). The *y*-axis represents distance from target coordinate (Y = 0) or % of cases in targeted area. Abbreviations: alv, alveus; CA1, CA1 subfield of the hippocampus; CA3, CA3 (CA3b, CA3c) subfield of the hippocampus; gcl, granule cell layer (s-gcl, suprapyramidal blade, i-gcl, infrapyramidal blade); hf, hippocampal fissure; l-m, stratum lacunosum moleculare of CA1; mol, molecular layer of the dentate gyrus; V, ventricle.

*EEG cohort.* The electrode tip was in the hippocampus or dentate gyrus in 82% (47/57) of the rats (Table 1). In the remaining 18% (10/57), the tip was located in either the fimbria, ventricle, dorsal thalamus, or out of the hippocampus (unidentified tip location) (Figure 7). The histologically verified AP and ML electrode tip locations were within 0.5 mm of the planned atlas coordinates in 65% (37/57) and 100% (56/56) of rats, respectively (Figure 7A,B). In one rat, the quality of the histological sections was not sufficient to determine the electrode location. The DV tip location was in the dentate gyrus in 54% (31/57) of rats. In the remaining 46% (26/57), the tip was located in either the CA1 or CA3 subfield of the hippocampus proper, fimbria, ventricle, or dorsal thalamus (Figure 7C).

*MRI cohort.* The electrode tip was in the hippocampus proper or dentate gyrus in 86% (36/42) of rats (Table 1). In the remaining 14% (6/42), the tip was located in either the ventricle or fimbria (Figure 7). The histologically verified AP and ML electrode tip locations were within 0.5 mm of the planned atlas coordinates of the MRI-guided location in 14% (6/43) and 100% (42/42) of rats, respectively (Figure 7A,B). The electrode tip was in the planned DV coordinate in 38% (16/42) of the rats. In the remaining 62% (26/42), the tip was located in either the hippocampal CA1 or CA3 subfields, hippocampal fissure, fimbria, or ventricle (Figure 7C).

#### 3.3.2. Anteroposterior Location of Hippocampal Electrode Tips

*EEG cohort.* Only 9% (5/57) of the electrode tips were in the planned atlas-based coordinate (AP: 3.0 mm). The remaining 77% (44/57) were positioned rostrally and 14% (8/57) were positioned caudally (Figure 7A). The mean deviation of the histologically verified coordinate from the atlas-based coordinate was 0.57 ± 0.07 mm (range: 0–2.52 mm, median: 0.48 mm). The deviation was comparable between sham-operated controls and TBI rats (0.54 ± 0.09 mm vs. 0.58 ± 0.09 mm; *p* > 0.05) (Figure 7D).

*MRI cohort.* The AP distribution of MRI-guided hippocampal electrode tip placements was anterior in 93% (39/42) and posterior in 7% (2/42) of the cases (Figure 7A). The average deviation of the histologically verified coordinate from the MRI-guided coordinate was 0.95 ± 0.08 mm (range: 0.04–2.28, median: 0.84) (Figure 7D). The deviation was comparable between sham-operated controls and TBI rats (0.66 ± 0.11 mm vs. 1.06 ± 0.10 mm; *p* > 0.05) (Figure 7D).

#### 3.3.3. Mediolateral Location of Hippocampal Electrode Tips

*EEG cohort.* The hippocampal electrode was located anterior to the atlas-defined target in 48% (27/56), medial in 50% (28/56), and on target in 2% (1/56) of the rats (Figure 5B). The mean deviation of the histologically defined coordinate from the atlas-defined coordinate was 0.21 ± 0.02 mm (range: 0–0.63 mm, median: 0.2 mm) (Figure 7D). The deviation was comparable between sham-operated controls and TBI rats (0.24 ± 0.04 mm vs. 0.19 ± 0.03 mm; *p* > 0.05) (Figure 7D).

*MRI cohort.* The histologically defined tip coordinate was lateral to the MRI-guided coordinate in 17% (7/42) and medial in 83% (34/42) of the rats. The average deviation of the histologically defined coordinate from the MRI-guided coordinate was 0.34 ± 0.04 mm (range: 0–0.9 mm, median: 0.3 mm) (Figure 7D). The deviation was comparable between sham-operated controls and TBI rats (0.35 ± 0.07 mm vs. 0.34 ± 0.05 mm; *p* > 0.05) (Figure 7D).

#### 3.3.4. Dorsoventral Location of Hippocampal Electrode Tips

*EEG cohort.* Histology revealed electrode path-associated lesions (see details in Section 3.4.2). In some TBI rats, the electrode path appeared slanted rather than vertical, probably due to hippocampal distortion related to enlarged ventricles.

The electrode tip was in the dentate gyrus in 49% (28/57) of the rats, locating in the molecular layer of the dentate gyrus in 17% (10/57) and in the granule cell layer in 32% (18/57) of the cases. No tips were observed in the hilus (Figure 7C). In the remaining rats, the tip was located in the hippocampus proper in 33% (5% [3/57] in the stratum lacunosum moleculare or hippocampal fissure, 5% [3/57] in CA1, 23% [13/57] in CA3), most of the tips in the CA3 subfield being in the CA3c subfield (Figure 5C). In 9% (5/57) of the cases, the tip was in the fimbria or ventricle. In 9% (5/57), the electrode went through the septal hippocampus and ended in the dorsal thalamus (*n* = 2) or in an unidentified location (*n* = 3) (see details in Section 3.4.2). The DV distribution of the electrode tips was comparable between sham-operated controls and TBI rats (ꭕ^2^ test; *p* > 0.05).

*MRI cohort.* Unlike in the EEG cohort, electrode path-associated lesions were small. Also, the electrode paths were vertical rather than slanted.

The electrode tip was in the targeted dentate gyrus in 36% (15/42) of animals, being 8% (3/42) in the molecular layer and 28% (12/42) in the granule cell layer (Figure 7C). No electrode tips were observed in the hilus. In 50% of the rats, the tip was in the hippocampus proper (7% [3/42] in the stratum lacunosum moleculare or hippocampal fissure, 5% [2/42] in CA1, and 38% [16/42] in CA3). Like in the EEG cohort, most of the tips in the CA3 were in the CA3c subfield (Figure 7C). In the remaining 14% (6/42), the tip was located in either the fimbria (*n* = 1) or the ventricle (*n* = 5) (Figure 7C). The DV distribution of tip locations was comparable between sham-operated controls and TBI rats (ꭕ^2^ test; *p* = 0.741).

### 3.4. Cortical and Hippocampal Atrophy after TBI

#### 3.4.1. Cortical Atrophy

**Figure 8:**
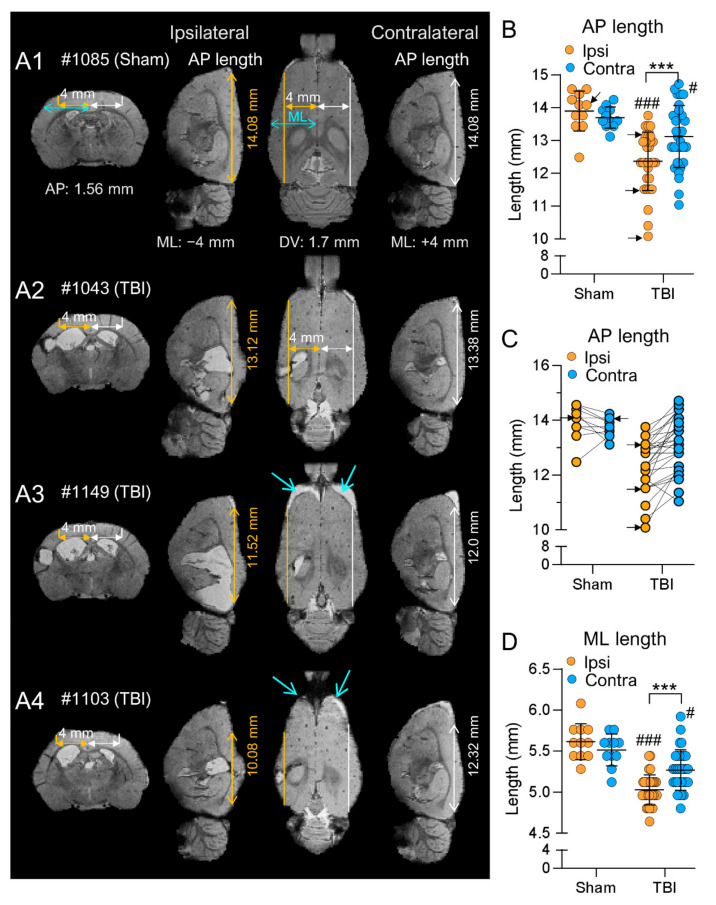
Anteroposterior and mediolateral shrinkage of the brain. In vivo magnetic resonance 3D multigradient echo (MGRE) images acquired at 5 months after TBI were used to estimate cortical shrinkage in the MRI cohort. (**A1**–**A4**) coronal, sagittal (ipsilateral and contralateral) and horizontal MGRE images of a sham rat (**A1**) and TBI rats (**A2**–**A4**). Anteroposterior (AP) cortical shrinkage was estimated by measuring the distance between the rostral and caudal cortical surface (double-headed arrows) in the sagittal slice at 4 mm from the midline both ipsilaterally (orange) and contralaterally (white). Note the change in the shape of the ipsilateral cortex (sagittal images) in TBI rats, indicating the TBI-induced cortical atrophy (see also turquoise arrows in (**A3**,**A4**)). Mediolateral (ML) shrinkage was assessed by measuring the distance between the midline and the lateral edge of the cortex (turquoise double headed arrow) in a horizontal slice at 1.7 mm below the pial surface at AP level −1.56 (corresponding to the targeted location of the anterior intracortical electrode tip). (**B**) A dot plot showing the ipsilateral (orange) and contralateral (blue) cortical AP lengths (*y*-axis) in the sham and TBI groups (*x*-axis). Note that both the ipsilateral and contralateral cortical AP lengths were reduced in TBI rats compared with sham-operated animals. Also, in the TBI group, the cortical AP length was shorter ipsilaterally than contralaterally. (**C**) A paired dot plot showing that the ipsilateral vs. contralateral shrinkage in each rat. The greater the ipsilateral shrinkage, the greater the contralateral shrinkage in the TBI compared with sham group. Arrows point to the 3 cases illustrated in panels (**A1**–**A4**). (**D**) A dot plot showing the ipsilateral and contralateral cortical ML lengths in sham-operated and TBI rats. Note that both the ipsilateral and contralateral cortical ML lengths were reduced in TBI rats compared with sham-operated animals. Also, in the TBI group, the cortical ML length was shorter ipsilaterally than contralaterally. Statistical significance: *** *p* < 0.001 compared with the contralateral hemisphere (Wilcoxon signed-rank test); ^###^ *p* < 0.001, ^#^ *p* < 0.05 compared with the sham group (Mann–Whitney *U* test).

*Anteroposterior.* In sham-operated controls, there was no difference in the cortical AP length at 4 mm lateral to the midline between the ipsilateral (13.89 ± 0.19 mm, range: 12.48–0.56 mm, median: 14.1 mm) and contralateral hemispheres (13.70 ± 0.09 mm, range: 13.12–14.24 mm, median: 13.6 mm; *p* > 0.05) (Figure 8A1,B,C).

In TBI rats, the cortical AP length was shorter ipsilaterally (12.37 ± 0.16 mm, range: 10.08–13.76 mm, median: 12.23 mm) than contralaterally (13.13 ± 0.17, range: 11.04–14.72 mm, median: 13.12 mm; *p* < 0.001) (Figure 8A2–A4,B). Both the ipsilateral (*p* < 0.001) and contralateral (*p* > 0.05) AP lengths were shorter in the TBI rats than in the sham group (Figure 8B,C).

*Mediolateral.* In sham-operated controls, the ML length was similar ipsilaterally (5.62 ± 0.06 mm, range: 5.28–6.08 mm, median: 5.6 mm) and contralaterally (5.51 ± 0.06 mm, range: 5.12–5.76, median: 5.6 mm; *p* > 0.05).

In the TBI group, the ML length was shorter ipsilaterally (5.03 ± 0.03 mm, range: 4.64–5.44, median: 4.96 mm) than contralaterally (5.27 ± 0.04 mm, range: 4.80–5.92 mm, median: 5.28 mm; *p* < 0.001). Both the ipsilateral (*p* < 0.001) and contralateral (*p* < 0.05) ML lengths were shorter in the TBI rats than in the sham group (Figure 8D).

#### 3.4.2. Hippocampal Atrophy

**Figure 9:**
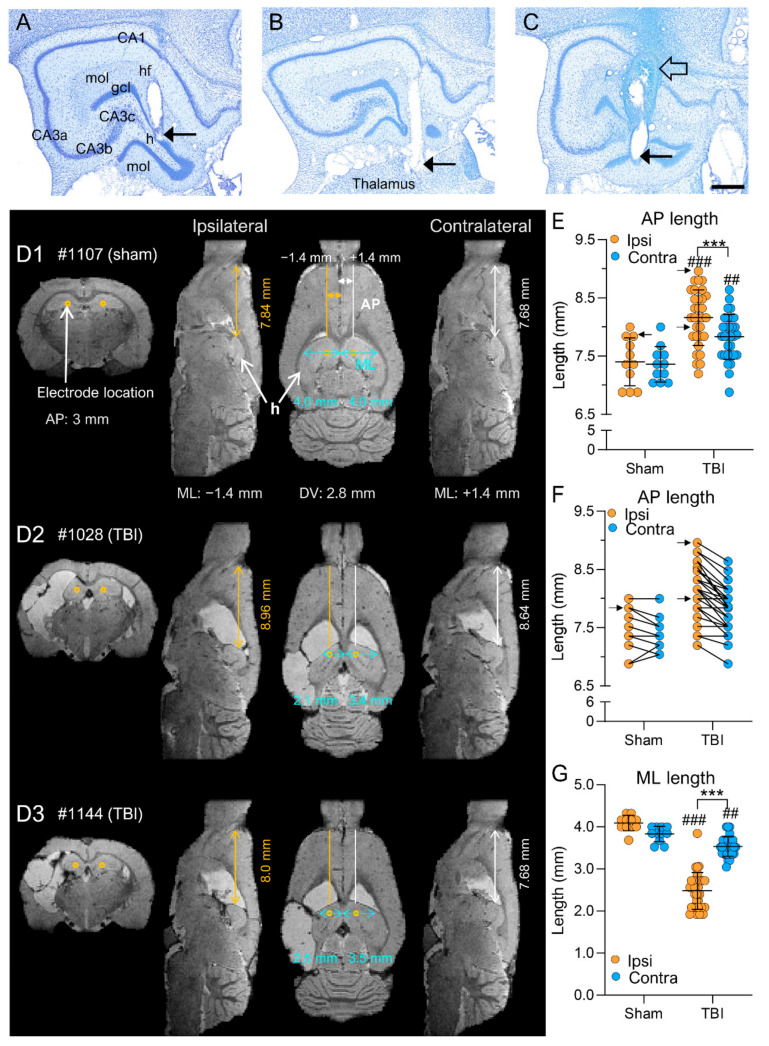
(**A**–**C**) Electrode tracts. Histological images from the coronal thionine-stained sections of 3 rats, showing the tracts of the bipolar intracortical electrodes and locations of the lower electrode tip (filled arrowhead). The target of the lower tip was the hilus. In panel (**A**), the electrode tip is in the suprapyramidal blade of the granule cell layer. In panel (**B**), the tip went through the dentate gyrus down to the dorsal thalamus. In panel (**C**), the tip is in the infrapyramidal blade of the granule cell layer. Note the electrode-path associated lesion in CA1 (open arrow). (**D1**–**D3**) Coronal, sagittal and horizontal in vivo magnetic resonance 3D multigradient echo (MGRE) images of the ipsilateral and contralateral hippocampus were used to assess hippocampal shrinkage after traumatic brain injury (TBI). (**D1**–**D3**) Hippocampal distortion and shrinkage. Panel (**D1**): A sham-operated experimental control (1107). Panels (**D2**,**D3**): Two rats with TBI (1028, 1144). The anteroposterior (AP) shift of the hippocampus was assessed by measuring the distance from the rostral edge of the frontal cortex to the rostral edge of the hippocampus at 1.4 mm from the midline in the horizontal slice 2.8 mm below the surface of the brain (left hemisphere: orange double-headed arrow; right hemisphere: white double-headed arrow). Mediolateral (ML) shrinkage was assessed by measuring the distance from the brain midline to the lateral edge of the hippocampus in the same horizontal plane (2.8 mm below the surface of the brain, turquoise double-headed arrow) in a slice sampled at AP level −2.8 mm, corresponding to the AP level of the atlas-based target coordinate. In both TBI rats (**D2**,**D3**), the distance from the frontal pole to the rostral edge of the hippocampus was longer than that in the sham-operated animal, indicating retraction of the septal hippocampus caudally. The ML length in TBI rats (**D2**,**D3**) was shorter than that in the sham-operated animal (**D1**), indicating a shift toward midline. (**E**) A dot plot showing the ipsilateral (orange) and contralateral (blue) anteroposterior lengths (*y*-axis) in the sham and TBI groups (*x*-axis). Note that both the ipsilateral and contralateral cortical AP lengths were increased in TBI rats compared with sham-operated animals. Also, in the TBI group, the cortical AP length was greater ipsilaterally than contralaterally. (**F**) A paired dot plot showing the ipsilateral vs. contralateral backward “movement” in each rat. The greater the ipsilateral “movement”, the greater the contralateral “movement”. Arrows point to the 3 cases illustrated in panels (**D1**–**D3**). (**G**) A dot plot showing the ipsilateral and contralateral hippocampal ML lengths in sham-operated and TBI rats. Note that both the ipsilateral and contralateral cortical ML lengths were reduced in TBI rats compared with sham-operated animals. Also, in the TBI group, the ML length was shorter ipsilaterally than contralaterally. Statistical significance: ^##^, *p* < 0.05; ^###^, *p* < 0.001 as compared to the sham group (Mann–Whitney U test); *** *p* < 0.001 compared with the contralateral hemisphere (Wilcoxon signed-rank test). Scale bar in (**A**–**C**) = 500 µm.

*Anteroposterior.* In the sham group, the average AP length was similar ipsilaterally (7.40 ± 0.12 mm, range: 6.88–8.0 mm, median: 7.36 mm) and contralaterally (7.36 ± 0.09 mm, range: 7.04–8.0 mm, median: 7.36 mm; *p* > 0.05) (Figure 9D1,E,F).

In the TBI group, the AP length was greater ipsilaterally (8.16 ± 0.09 mm, range: 7.2–8.96 mm, median: 8.16 mm) than contralaterally (7.83 ± 0.07 mm, range: 6.88–8.64 mm, median: 7.84 mm; *p* < 0.001) (Figure 9D2,D3,E,F). Both the ipsilateral (*p* < 0.001) and contralateral (*p* < 0.01) AP lengths were greater in the TBI rats than in the sham group.

*Mediolateral.* In the sham group, the ML length was comparable ipsilaterally (4.09 ± 0.05 mm, range: 3.68–4.32 mm, median: 4.16 mm) and contralaterally (3.83 ± 0.06 mm, range: 3.52–4.0 mm, median: 3.84 mm; *p* > 0.05) (Figure 9D1,G).

In the TBI group, the ML length was shorter ipsilaterally (2.48 ± 0.08 mm, range: 1.92–3.84 mm, median: 2.4 mm) than contralaterally (3.53 ± 0.04 mm, range: 3.04–4.0 mm, median: 3.52 mm; *p* < 0.001) (Figure 7G and Figure 9D2,D3). Both ipsilateral (*p* < 0.001) and contralateral (*p* < 0.01) ML lengths were shorter in the TBI rats than in the sham group (Figure 9).

### 3.5. “Virtual Electrode”—Comparison of Success Rate in Atlas-Based vs. MRI-Guided Electrode Placements

Finally, to assess whether the MRI images indeed improved the targeting of the electrode tip to the perilesional cortex, and not, for example, to the lesion cavity, we reexamined the histological sections of TBI rats in the MRI cohort. We focused on the caudal aspect of the brain, as targeting this area without the use of MRI was challenging due to remarkable TBI-related cortical atrophy.

**Figure 10:**
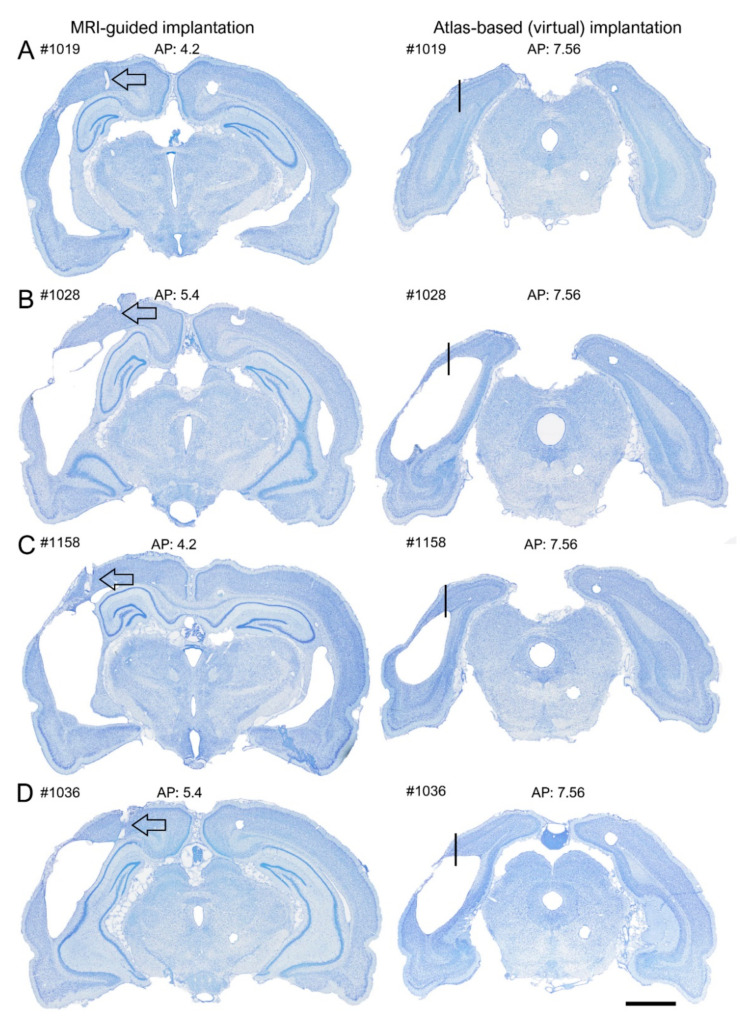
Location of electrode tip at 5 months postinjury without prior MRI analysis. Photomicrographs of thionine-stained coronal brain sections of 4 animals; (**A**) #1019, (**B**) #1139, (**C**) #1158 and (**D**) #1036 in the MRI cohort with electrode implantations at 5 months after TBI. Left panels: MRI-guided placement of the posterior cortical electrode. Note that all electrodes are within the perilesional cortex. Right panels: The location of the electrode tip (arrow), if the electrode was implanted according to the targeted atlas-based coordinates (−7.56 mm from bregma, 4 mm from midline, 1.8 mm from the surface of the brain). Note that in all rats except 1019, the electrode tip ended in the lesion cavity. Table 2 summarizes the locations for all cases. Scale bar = 2 mm.

A hypothetical “virtual” electrode was placed at the atlas-defined coordinate of the posterior intracortical electrode (Figure 10). We then reconstructed the destination of the electrode tip in the available histological sections by assessing (a) whether it was located in the cortex or lesion cavity and (b) the distance of the tip from the lesion edge. We found that by using the atlas-based coordinate (AP −7.56), 58% (18/31) of the electrodes had been in the lesion cavity compared with 0% for the MRI-guided implantations (Figure 5C and Figure 10 and Table 2). The remaining 42% (13/31) of the “virtual” electrodes were located medial to the lesion cavity, except in one case (rat 1103), in which the tip location was caudal to the lesion. The average distance of the electrode tip to the lesion edge was 0.64 ± 0.1 mm (range: 0–1.3 mm) (see also Supplementary Table S2 for further details).

## 3.6. Effect of Electrode Implantation on Progression of the Cortical Lesion

Next, we assessed whether a 6-month-long presence of intracortical electrodes enhanced cortical atrophy. We hypothesized that (1) the lesion area would be greater in the EEG cohort than in the MRI cohort and (2) the cortical electrode tips would be closer to the lesion cavity (expected distance ≥ 500 µm) in the EEG cohort than in the MRI cohort.

**Figure 11:**
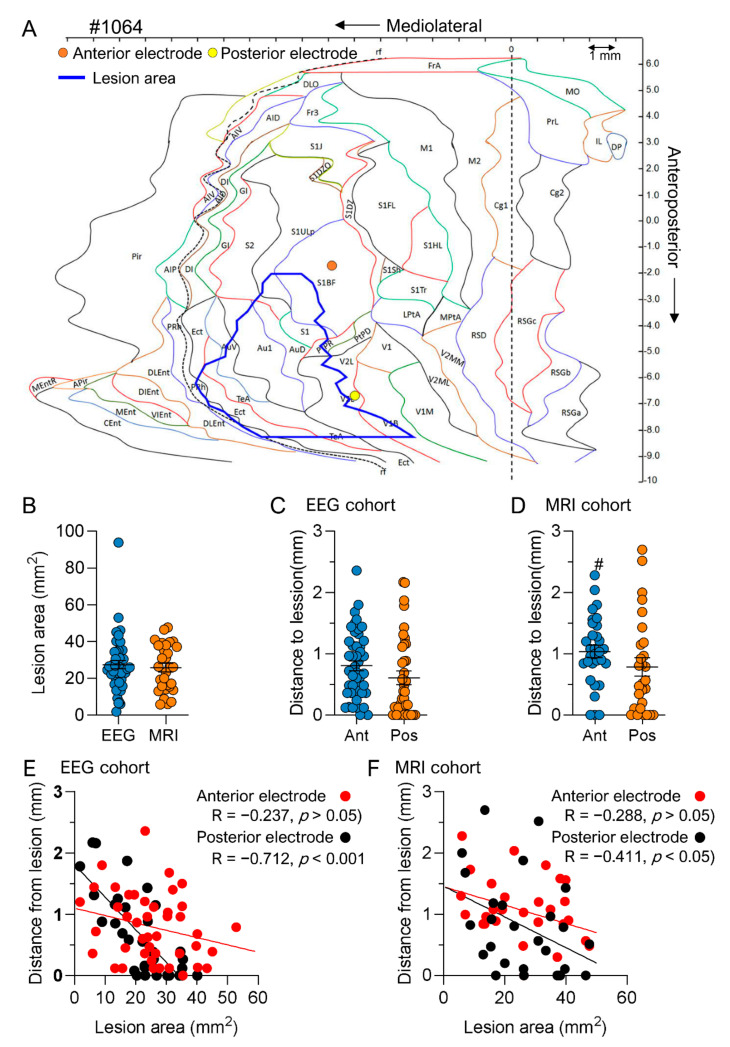
Distance of the intracortical electrodes from the edge of the cortical lesion cavity. (**A**) An unfolded cortical map of a rat 1064, showing the cytoarchitectonic distribution of the cortical lesion (blue outline) and the location of the anterior (brown filled circle in the S1BF) and posterior (yellow filled circle in the V2L) intracortical electrodes. Note that the lesion had progressed laterally and caudally. Consequently, the posterior electrode was closer to the lesion cavity edge than the anterior electrode. (**B**) A scatter plot showing the cortical lesion area in the EEG and MRI cohorts (each dot represents 1 rat). The lesion area was comparable between cohorts (*p* > 0.05). (**C**) In the EEG cohort, the distance from the electrode tip to the lesion cavity edge (layer V intersection was used as reference) was similar between the anterior and posterior intracortical electrodes (*p* > 0.05). (**D**) In the MRI cohort, the distance from the anterior intracortical electrode tip to the cavity edge was slightly greater than that from the EEG cohort (*p* < 0.05). (**E**) In the EEG cohort, the larger the lesion, the closer the posterior electrode tip to the lesion cavity edge (*p* < 0.001). (**F**) In the MRI cohort, the larger the lesion, the closer the posterior electrode tip to the cavity edge (*p* < 0.001). Abbreviations: S1BF, primary somatosensory barrel field; V2L, secondary visual cortex lateral area. Statistical significance: ^#^, *p* < 0.05 compared with the EEG cohort (Mann–Whitney U test).

*Lesion area and location.* The cortical lesion spread laterally and caudally, typically involving the sensory, auditory, and visual cortices, as previously described [32] (Figure 11A). The average total lesion area was comparable between the EEG (27.31 ± 2.29 mm^2^, range: 1.72–93.8 mm^2^, median: 25.8 mm^2^) and MRI (25.69 ± 2.32 mm^2^, range: 5.7–47.6 mm^2^, median: 26.1 mm^2^) cohorts (*p* > 0.05) (Figure 11B). Also, the mean lesion area in the primary somatosensory (4.89 ± 0.41 mm^2^ vs. 5.16 ± 0.55 mm^2^; *p* > 0.05) and visual cortex (7.73 ± 0.72 mm^2^ vs. 7.37 ± 0.51 mm^2^; *p* > 0.05) was similar in the EEG and MRI cohorts (Table 3). In the secondary somatosensory cortex (S2) (0.56 ± 0.07 mm^2^ vs. 1.07 ±0.17 mm^2^; *p* < 0.01) and the primary auditory cortex (Au1) (3.77 ± 0.20 mm^2^ vs. 4.32 ± 0.30 mm^2^; *p* < 0.05) the lesion area was smaller in the EEG cohort than in the MRI cohort (Table 3).

*Electrode distance from the lesion cavity.* In the EEG cohort, 54% of the anterior intracortical electrodes were located anterior to the rostral edge of the lesion and 46% were located medial to the lesion. The average distance to the lesion edge was 0.79 ± 0.08 mm (range: 0–2.36 mm, median: 0.71 mm) (Figure 11C). All posterior intracortical electrodes were located medial to the lesion; 33%, however, were observed on the edge of the lesion or in the lesion cavity. The mean distance to the lesion edge was 0.63 ± 0.11 mm (range: 0–2.17 mm, median: 0.38 mm), comparable to that of the anterior intracortical electrode (*p* > 0.05).

In the MRI cohort, 41% of the anterior intracortical electrodes were located anterior to the rostral edge of the lesion and 59% were located medial to the lesion. The distance to the lesion edge was greater in the MRI (1.038 ± 0.10 mm, range: 0–2.3 mm, median: 1.03 mm) than in the EEG cohort (*p* < 0.05). All posterior intracortical electrodes (100%) were located medial to the lesion. Unlike in the EEG cohort, none of the tips was at the lesion edge or in the cavity. The distance of the posterior electrode tip to the lesion edge (0.79 ± 0.15 mm, range: 0–2.7 mm, median: 0.54 mm) was comparable to that of the anterior intracortical electrode (*p* > 0.05) (Figure 11D) or the posterior intracortical electrode in the EEG cohort (*p* > 0.05).

In both cohorts, we were unable to verify the hypothesis that the greater the lesion, the closer the tip of the anterior intracortical electrode to the lesion edge as there was no correlation between lesion size and the distance of the anterior intracortical electrode from the lesion edge (EEG cohort: R = −0.237; *p* > 0.05; MRI cohort: R = −0.288; *p* > 0.05) (Figure 9E,F). In the case of posterior cortical electrodes, however, the larger the lesion, the closer the tip to the lesion edge (EEG cohort: R = −0.712; *p* < 0.0001, MRI cohort: R = −0.411; *p* < 0.05) (Figure 9E,F).

## 4. Discussion

To address the challenges related to chronic implantation of electrodes in brain-damaged rats, our objective was to develop methodologies to maximize the accuracy of chronic recording-electrode placements using preimplantation structural MRI. In the material available, we also assessed the effect of chronic electrode implantations on TBI-induced brain atrophy. Our data revealed that (1) animal-dependent progression of the cortical lesion after TBI compromises the placement accuracy of depth electrodes implanted at later time-points when only atlas-based coordinates are used; (2) MRI-guided adjustment of atlas-based coordinates increases the placement accuracy of intracerebral electrodes at the chronic post-TBI phase, particularly in the perilesional cortex; and (3) chronically implanted electrodes do not increase cortical and/or hippocampal atrophy.

### 4.1. Electrode Implantation Immediately after TBI Resulted in Good Location Accuracy of Intracerebral Electrode Tips Rostrally, but Was Less Accurate Caudally

In the EEG cohort, we implanted two bipolar intracortical and one bipolar hippocampal electrode ipsilateral to the lesion immediately after TBI to monitor the acute post-TBI electrophysiologic events and followed up the evolution of epileptiform activities over the following 7 months [36,37]. We assumed that the deformation and atrophy of the brain were not compromising the electrode placement accuracy at this early postinjury time-point, and thus we could rely on the rat brain atlas designed for the normal brain in defining the AP and DV coordinates for electrode placements. The fixed atlas-based cortical and hippocampal AP coordinates were chosen based on our previous observations that the cortical lesion progresses laterally and caudally [32]. In particular, it is critical to position the electrode tip in the anticipated epileptogenic area in the perilesional cortex, but avoid the lesion cavity [13].

Despite the unpredictable caudal progression of the cortical lesion, 83% of the anterior and 40% of the posterior cortical electrodes were within the cortex, and there was no difference in the overall DV distribution of the electrode tips between sham-operated controls and TBI rats. In 10% of the animals, however, the electrode tip of the posterior cortical electrodes had entered the lesion cavity when assessed at 7 months after implantation. This was particularly evident in cases with a large lesion size, which associated with robust cortical thinning. Also, 10% of the posterior cortical electrodes had entered the hippocampus, and consequently, recorded hippocampal EEG instead of cortical EEG. Progression of cortical lesions in the lateral FPI model is known to be variable and unpredictable [7,21,32,38]. It is possible that the progressive cortical atrophy “melting” of the brain around the electrode tips may have caused many of the electrode tips to end in the lesion cavity or subcortical areas, including the angular bundle and hippocampus. Even in the presence of an acute preimplantation MRI of the rats included in the EEG cohort, it was difficult to estimate the correct location for the intracerebral cortical electrodes 7 months later.

Although we were quite successful in implanting the electrode tips in the cortical tissue, both the anterior and posterior electrodes tended to locate slightly more anterior than planned. The anterior deviation from the target was typically less than 1 mm, however, and the mediolateral deviation was even smaller. We assume that the use of the Sprague Dawley strain in the present experiments instead of Wistar rats, which were used in the atlas preparation, and also mild human errors in reading bregma, could have affected the accuracy of the electrode placements. It is also important to note that depending on the skull size, the location of the 5 mm-diameter craniotomy slightly varied, which could also affect electrode insertion.

The target of the lower hippocampal electrode tip was at the hilus of the dentate gyrus. Consequently, the upper tip was expected to be in the CA1 subfield. As such, we aimed to have at least one electrode tip recording hippocampal epileptiform activity. Although in previous studies we noticed the development of hippocampal atrophy and deformations over months postinjury, we had no quantitative data to estimate the adjustments needed to fix the atlas-guided electrode tip coordinates. Somewhat disappointingly, at 7 months after the electrode implantation in the EEG cohort, the electrode tip was outside the dentate gyrus in half of the cases. In the remaining cases, the electrode tips were mainly in the CA3c subfield of the hippocampus proper. Only 7% of the electrodes were outside the hippocampus and not recording. In two cases, the electrodes were recording in the thalamus. We assume that postimpact subdural hematoma and/or edema affected the reading of the pial surface, which is used to calculate the DV coordinate, leading to mild misplacement of the hippocampal electrode in the TBI rats, particularly as the CA3c misplacement was observed in 1 sham rat only. Moreover, the implantation was technically challenging, as the AP coordinate of the hippocampal electrode was very close to the rostromedial edge of the craniotomy, leading in some cases to more rostral electrode repositioning of the AP coordinate planned during the surgery.

Taken together, our data show that the location of intracortical electrodes implanted immediately after TBI in the lateral FPI model, particularly to the posterior parts of the brain, can be compromised by unpredictable caudolateral progression of the cortical lesion and cortical thinning, leading to electrode misplacement affecting the EEG recordings. The hippocampal electrode placements immediately after TBI for chronic EEG follow-up are generally more stable and less affected by post-TBI hippocampal morphologic transformations; even the acute cortical swelling and subdural hematoma can affect DV electrode placement. As 30% of the 297 intracortical or hippocampal electrodes were recording outside the target tissue (cortex or hippocampus/dentate gyrus), verification of the electrode tip locations is needed for accurate interpretation of chronic EEG recordings even when the electrode implantations are performed in the early postinjury time period.

### 4.2. Preimplantation Structural MRI Improved the Accuracy of Electrode Implantations at 5 Months Postinjury

The interim review of MRIs imaged during the 5-month follow-up of animals in the MRI cohort confirmed our previous observations of the progression of cortical lesion in injured rats and raised concerns about the accuracy of electrode placements for the 1-month 24 h/7-day high-density video-EEG recordings, which was critical for the epilepsy phenotyping in our animal cohorts. It is generally recognized that proper electrode placement is a basis for accurate EEG data interpretation. To maximize the success rate of electrode implantations, we used images of coronal in vivo MR T2-wt slices obtained at 5 months post-TBI to generate rat-specific AP, ML, and DV coordinates for the anterior and posterior cortical electrodes, as well as the hippocampal electrodes. The aim was to place the cortical electrodes in the perilesional cortex within 500 µm of the lesion cavity, similar to that in the EEG cohort. Moreover, we wanted to get the DV coordinate of the ventral electrode tip to layer V of the sometimes very atrophied cortex. As in the EEG cohort, the hippocampal electrode tip was targeted to the dentate gyrus.

Analysis of histological sections prepared from the same rats approximately 1 month after the MRI, i.e., right after finishing the 1-month video-EEG monitoring, revealed a rostral shift of both the cortical and hippocampal electrodes relative to the MRI-guided coordinate. These data suggest that cortical atrophy, retracting the brain backwards may have contributed to the anterior shift and low accuracy of the cortical electrodes, particularly the posterior cortical electrode, in injured animals [7,17,39]. In case of hippocampal electrodes, the anterior shift was clearer in TBI rats than in sham animals, similar to the EEG cohort. Even though deviation of the hippocampal electrodes from the planned position can also relate to hippocampal atrophy, changes in its septotemporal orientation, rotation, and medial shift toward midline can contribute to electrode misplacements [23].

Generally, despite the unexpected divergence between the MRI-defined and histologically verified “true” coordinates, our data demonstrate that the MR images were useful when targeting the perilesional cortex for EEG recordings during the chronic phase post-TBI. Our “simulation” revealed that most (60%) of the MRI-guided posterior cortical electrode placements were in the cortex compared with 42% of the posterior cortical electrode placements when only the atlas-based coordinate was used. Moreover, with MRI-guided implantation, we were able to avoid the cortical lesion cavity. Additionally, the effect of cortical thinning on the DV location of the posterior electrode was mitigated, as the percentage of electrodes in the cortex was comparable between sham and TBI rats. Moreover, there was a 40% increase in cases with a cortical electrode location and a 14% increase in reaching the target layer V compared with the EEG cohort. We also demonstrated that despite the inaccuracy in adjusting the hippocampal AP coordinate when using MRI guidance, 36% of the hippocampal electrodes were recording in the dentate gyrus in the MRI cohort compared with 49% in the EEG cohort. This finding suggests that a 3D change in hippocampal orientation, which was less evident in the 2D MR images used in this study, compromised the estimation of the hippocampal AP coordinate. The 2D images were effective for determining the DV hippocampal target as the overall DV distribution of the electrode tip in the dentate gyrus was the same between sham and TBI rats.

One question is: Would presurgery MRIs help to improve the interpretation of postsurgery MRIs and the accuracy of electrode placements? In addition to the cost, it is important to note that unlike MRI, the histology-based rat brain atlas offers a benefit of using skull landmarks for calculation of coordinates for electrode positioning, that is, using the bregma as a reference point. Histological sections also give a higher spatial resolution and possibility to also assess the laminar placement of electrode tips.

Taken together, MR images were useful in targeting the AP and DV locations of perilesional intracortical electrodes in the chronic post-TBI phase. For hippocampal electrode implantations, the MR images were not as useful in targeting the AP location, but improved the precision of targeting the DV electrode tip into the dentate gyrus. To increase the accuracy of estimating the AP coordinate for intracerebral electrode implantation at the chronic post-TBI phase, we suggest using a 3D reconstruction of the brain to fully understand the effect of the ipsilateral cortical and hippocampal atrophy and orientation affecting the electrode placements. We propose the following MRI protocol for estimating the adjustments needed for atlas-based coordinates to successfully and accurately implant electrodes in the chronic post-TBI phase (see also Supplementary Figure S1):

1. Acquire T2-weighted MRI and 3D MGRE datasets.
2. Select the coronal T2-weighted and/or MGRE images containing the target region of the intracerebral electrode (e.g., perilesional cortex or hippocampus).
3. In each MGRE image selected in step 2, calculate the ML distance of the intended electrode tip location (e.g., perilesional cortex). Select the sagittal MGRE image at that ML coordinate. In MGRE sagittal images, calculate the DV distance (from the brain surface) to which the tip of the electrode will be targeted, and select the MGRE horizontal images at that depth.
4. In the sagittal and horizontal images selected in step 3, estimate the cortical AP (in the sagittal plane) and ML (in the horizontal plane) distances (shrinkage) for a given rat as described in this study, using available 3D slice viewer and analysis software
5. Determine the ratio of the AP distance based on the atlas coordinates/AP distance measured in the sagittal slice in a given rat (estimated in step 4).
6. To determine the final MRI AP coordinate, align the selected coronal images (step 2) with the atlas. Multiply the atlas AP coordinate at this location with the ratio determined in step 5.
7. To determine the final MRI ML location, repeat steps 5 and 6 in the horizontal plane.

### 4.3. Chronic Intracerebral Electrode Implantation Did Not Affect the Cortical Lesion Area or Cortical and Hippocampal Atrophy over the 7-Month Follow-Up

Chronic electrode implantation has been proposed to add tissue damage due to blood–brain barrier damage and chronic inflammation in the electrode path [40]. Therefore, we expected to see more cortical atrophy in the EEG than the MRI cohort. The cortical lesion areas were comparable in the EEG and MRI cohorts. Also, the percentage of the lesion area in cytoarchitectonic areas targeted by the electrode tips did not differ between the EEG cohort and MRI cohort. Taken together, the progression of the cortical lesion in the EEG cohort was not augmented by the chronically implanted electrodes.

We recently demonstrated that the hippocampus undergoes a series of post-TBI morphologic transformations, including atrophy and orientation changes due to neurodegeneration, white-matter atrophy, and expanding ventricles [23]. The present analysis revealed a caudal shift of the hippocampus. Apparently, the expanding ventricles pushed the hippocampus backward and toward the midline.

## 5. Conclusions

Our study demonstrates the benefit of using MR images for adjusting atlas-based coordinates to improve the accuracy in chronic intracerebral electrode placements into atrophied and distorted brain areas, which is critical, e.g., for a high-quality recording of various epileptiform activities in candidate epileptogenic regions after TBI. Future studies should consider using T2-wt MRI slices much thinner than 800 µm or 3D spatial encoding with close to isotropic resolution to allow for accurate visualization in coronal, sagittal, and horizontal planes to enhance the accuracy of MRI-guided electrode implantations. Importantly, comparison of the cortical lesion area and cytoarchitectonic distribution between cohorts with 7-month- or 1-month-long electrode implantations did not reveal any worsening of brain atrophy by the chronic electrode implantation.

## Figures and Tables

**Table 1:** Summary of the location of the dorsoventral tip of the intracerebral electrodes.

	EEG Cohort Atlas-Based % (*n*)			MRI Cohort Atlas-Based without MRI Guidance % (*n*)			MRI Cohort with MRI Guidance % (*n*)			EEG and MRI Cohorts % (*n*)		
	Sham	TBI	All	Sham	TBI	All	Sham	TBI	All	Sham	TBI	All
***Anterior cortical electrode***												
Within 1 mm of target coordinate ^#^	29% (4/14)	26% (11/43)	26% (15/57)	100% (11/11)	100% (31/31)	100% (42/42)	55% (6/11)	32% (10/31)	38% (16/42)	42% (10/25)	28% (21/74)	31% (31/99)
In cortex	79% (11/14)	84% (36/43)	83% (47/57)	100% (11/11)	32% (10/31)	50% (21/42)	73% * (8/11)	77% *** (24/31)	76% (32/42)	76% (19/25)	81% (60/74)	78% (79/99)
In brain	100% (14/14)	100% (43/43)	100% (57/57)	100% (11/11)	52% (16/31)	64% (27/42)	100% (11/11)	100% *** (31/31)	100% (42/42)	100% (25/25)	100% (74/74)	100% (99/99)
Not recording in brain	0% (0/14)	0% (0/43)	0% (0/57)	0% (0/111)	48% (15/31)	36% (15/42)	0% (0/11)	0% *** (0/31)	0% (0/42)	0% (0/25)	0% (0/74)	0% (0/99)
***Hippocampal electrode***												
Within 1 mm of target coordinate ^#^	93% (13/14)	77% (33/43)	81% (46/57)	100% (11/11)	100% (31/31)	100% (42/42)	82% (9/11)	52% (16/31)	60% (25/42)	88% (22/25)	66% (49/74)	72% (71/99)
In hippocampus proper/DG	86% (12/14)	81% (35/43)	82% (47/57)	100% (11/11)	100% (31/31)	100% (42/42)	100% (11/11)	81% * (25/31)	86% (36/42)	92% (23/25)	81% (60/74)	84% (83/99)
In brain	93% (13/14)	93% (41/43)	95% (54/57)	100% (11/11)	100% (31/31)	100% (42/42)	100% (11/11)	84% * (26/31)	88% (37/42)	96% (24/25)	91% (67/74)	92% (91/99)
Not recording in brain	7% (1/14)	5% (2/43)	5% (3/57)	0% (0/111)	0% (0/31)	0% (42/42)	0% (0/11)	16% * (5/39)	12% (5/42)	28% (7/25)	10% (7/74)	8% (8/99)
***Posterior cortical electrode***												
Within 1 mm of target coordinate ^#^	43% (6/14)	51% (20/39)	49% (26/53)	100% (11/11)	100% (31/31)	100% (42/42)	36% (4/11)	21% (6/29)	25% (10/40)	40% (10/25)	38% (26/68)	41% (38/93)
In cortex	21% (3/14)	46% (18/39)	40% (21/53)	91% (10/11)	42% (13/31)	55% (23/42)	46% * (5/11)	66% * (19/29)	60% (24/40)	32% (8/25)	54% (37/68)	48% (45/93)
In brain	100% (14/14)	97% (34/35)	90% (48/53)	100% (11/11)	42% (13/31)	57% (24/42)	100% (11/11)	93% *** (27/29)	95% (38/40)	100% (25/25)	90% (61/68)	92% (86/93)
Not recording in brain	0% (0/14)	13% (5/39)	10% (5/53)	0% (0/11)	58% (18/31)	43% (18/42)	0% (0//11)	7% *** (2/29)	5% (2/40)	0% (0/25)	10% (7/68)	8% (7/93)

^#^ Within <1 mm radius from the atlas-planned anteroposterior and mediolateral coordinate. The success rate was evaluated based on the location of the lower tip of bipolar electrode. Abbreviations: DG, dentate gyrus**.** Statistical significance: * *p* < 0.05; *** *p* < 0.001 compared to MRI cohort atlas-based without MRI guidance (ꭕ^2^ test).

**Table 2:** Location of the “virtual electrode”. Summary of the locations of the posterior intracortical electrodes in the MRI cohort, if implanted according to the atlas-based coordinates. Note that 58% (18/31) of the lower electrode tips were in the cortex while 42% (13/31) of the lower tips were in the lesion cavity. After MRI-guidance, 71% (22/31) of the lower tips were recording in the cortex. In 5 additional cases, the upper tip (0.5 dorsal to the lower tip) was expected to record in the cortex, resulting in a total of 87% (27/31) of the electrodes recording in the cortex. Only 1 electrode was not recording in the brain.

	Atlas-Based		MRI-Guided	
Animal	AP Level	DV Location (Distance from Lesion Edge)	AP Level	DV Location
1008	7.56	Lesion cavity	−4.68	Layer VI
1012	7.56	Lesion cavity	−5.20	CA1
1019	7.56	Perilesional cortex (medial, 0 mm)	−4.20	Layer V
1024	7.56	Lesion cavity	−4.68	angular bundle
1028	7.56	Lesion cavity	−4.36	Layer VI
1029	7.56	Lesion cavity	−4.36	corpus callosum
1031	7.56	Lesion cavity		not found
1036	7.56	Lesion cavity	−5.28	Layer VI
1038	7.56	Perilesional cortex (medial, 0.7 mm)	−4.68	alveus of the hippocampus
1043	7.56	Lesion cavity	−3.96	Layer IV
1046	7.56	Lesion cavity	−4.08	Layer V
1090	7.56	Perilesional cortex (medial, 0.53 mm)	−4.36	Layer VI
1095	7.56	Perilesional cortex (medial, 0.32 mm)	−3.96	Layer VI
1099	7.56	Perilesional cortex (medial, 0.93 mm)	−4.20	Layer VI
1103	7.56	Perilesional cortex (caudal, 0.2 mm)	−4.20	Layer V
1104	7.56	Perilesional cortex (medial, 0.73 mm)	−5.28	Layer V
1105	7.56	Lesion cavity	−4.68	Layer VI
1138	7.56	Perilesional cortex (medial, 0.71 mm)	−3.96	Layer V
1139	7.56	Lesion cavity	−5.40	Layer V
1140	7.56	Perilesional cortex (medial, 1.1 mm)	−5.52	Layer VI
1142	7.56	Lesion cavity	−4.20	corpus callosum
1144	7.56	Lesion cavity	−4.80	ventricle
1145	7.56	Perilesional cortex (medial, 0.63 mm)	−4.20	CA1
1149	7.56	Lesion cavity	−4.20	Layer VI
1150	7.56	Lesion cavity	−4.20	Layer VI
1152	7.56	Perilesional cortex (medial, 0.91 mm)	−4.68	Layer V
1153	7.56	Lesion cavity	−4.20	Layer VI
1154	7.56	Lesion cavity	−5.40	Layer VI
1156	7.56	Perilesional cortex (medial, 0.28 mm)	−4.68	Layer VI
1158	7.56	Lesion cavity	−4.20	Layer VI
1159	7.56	Perilesional cortex (medial, 1.3 mm)		not found

**Table 3:** Cytoarchitectonic distribution of the TBI-induced cortical lesion in the EEG and MRI cohorts.

	EEG Cohort			MRI Cohort		
Cortical Area	Area (mm^2^) (Mean ± SEM)	Min–Max	% of Rats	Area (Mean ± SEM)	Min–Max	% of Rats
DLEnt	0.27 ± 0.01	0.27–0.27	5 (2/43)			0 (0/30)
PRh	0.44 ± 0.11	0.01–1.35	44 (19/43)	0.79 ± 0.16	0.10–1.61	37 (11/30)
Ect	1.47 ± 0.20	0.01–3.82	77 (33/43)	1.77 ± 0.33	0.08–5.11	70 (21/30)
DI			0 (0/43)	0.18	0.18–0.18	3 (1/30)
GI			0 (0/43)	0.12 ± 0.05	0.03–0.24	13 (4/30) ^#^
TeA	2.35 ± 0.17	0.01–3.92	91 (39/43)	2.33 ± 0.26	0.01–3.92	87 (26/30)
AuV	0.91 ± 0.09	0.01–2.45	93 (40/43)	1.22 ± 0.15	0.02–2.62	90 (27/30)
Au1	3.77 ± 0.20	0.86–5.78	100 (43/43)	4.32 ± 0.30 *	0.66–5.86	100 (30/30)
AuD	2.31 ± 0.06	0.58–2.61	100 (43/43)	2.32 ± 0.09	1.04–2.61	100 (30/30)
S1ULp	0.75 ± 0.09	0.03–1.98	93 (40/43)	1.14 ± 0.12 *	0.13–2.52	80 (24/30)
S1	1.16 ± 0.03	0.49–1.23	98 (42/43)	1.09 ± 0.05	0.02–1.23	97 (29/30)
S1BF	3.79 ± 0.36	0.56–10.44	98 (42/43)	3.42 ± 0.41	0.16–8.68	93 (28/30)
S1DZ	0.19 ± 0.06	0.02–0.3	9 (4/43)	0.01	0.01–0.01	3 (1/30)
PtPR	0.61 ± 0.03	0.06–0.75	95 (41/43)	0.61 ± 0.04	0.01–0.75	97 (29/30)
PtPD	0.47 ± 0.05	0.01–0.97	86 (37/43)	0.47 ± 0.05	0.01–0.96	83 (25/30)
V2L	4.62 ± 0.27	0.02–6.28	100 (43/43)	4.60 ± 0.32	0.87–6.48	100 (30/30)
V1B	2.12 ± 0.23	0.02–4.59	81 (35/43)	2.19 ± 0.30	0.15–4.81	80 (24/30)
V1	0.73 ± 0.16	0–2.13	56 (24/43)	0.37 ± 0.09	0.05–1.21	60 (18/30)
V1M	1.42 ± 0.26	0.01–3.98	47 (20/43)	1.32 ± 0.28	0.12–3.54	53 (16/30)
V2ML	0.68 ± 0.29	0.03–2.7	21 (9/43)	0.24 ± 0.14	0.01–0.76	17 (5/30)
V2MM	0.76 ± 0.50	0.04–2.26	9 (4/43)	0.28 ± 0.15	0.02–1.01	20 (6/30)
MPtA	0.04 ± 0.02	0.02–0.06	5 (2/43)			0 (0/30)
LPtA	0.83 ± 0.22	0.06–1.62	16 (7/43)	0.04 ± 0.03	0.01–0.09	10 (3/30)
S1Tr	0.54 ± 0.27	0.2–1.09	7 (3/43)			0 (0/30)
S1Sh	0.01	0.01–0.01	2 (1/43)			0 (0/30)
S1FL	0.12	0.12–0.12	2 (1/43)			0 (0/30)
S2	0.56 ± 0.07	0.04–2.10	95 (41/43)	1.07 ± 0.17 *	0.38–3.09	80 (24/30) ^#^
RSD	1.09 ± 0.91	0.17–2.0	5 (2/43)	0.20 ± 0.08	0.02–0.42	13 (4/30)
RSGc	0.37	0.37–0.37	2 (1/43)			0 (0/30)
Sum of S1	4.89 ± 0.41	0.00–12.09	(43/43)	5.16 ± 0.55	0.00–11.55	(30/30)
Total S	5.43 ± 0.44	0.00–13.22	(43/43)	6.01 ± 0.65	0.00–12.59	(30/30)
Sum of V1	2.80 ± 0.46	0.00–10.20	(43/43)	2.68 ± 0.49	0.00–9.12	(30/30)
Sum of V2	4.84 ± 0.31	0.02–10.76	(43/43)	4.69 ± 0.34	0.87–7.71	(30/30)
Total V	7.63 ± 0.72	0.02–20.95	(43/43)	7.37 ± 0.78	0.87–15.82	(30/30)
Total Au	6.93 ± 0.34	1.44–10.84	(43/43)	7.73 ± 0.51	1.86–11.08	(30/30)
Total Area	25.33 ± 1.74	1.72–52.88	(43/43)	25.69 ± 2.32	5.66–47.61	(30/30)

Abbreviations: Au1, primary auditory cortex; AuD, secondary auditory cortex, dorsal area; AuV, secondary auditory cortex, ventral area; DLEnt, dorsolateral entorhinal cortex; DI, dysgranular insular cortex; Ect, ectorhinal cortex; GI, granular insular cortex; LPtA, lateral parietal association cortex; MPtA, medial parietal association cortex; PRh, perirhinal cortex; PtPD, parietal cortex, posterior area, dorsal part; PtPR, parietal cortex; RSD, retrosplenial dysgranular cortex; RSGc, retrosplenial granular cortex, c region; S1, primary somatosensory cortex; S2, secondary somatosensory cortex; S1BF, primary somatosensory cortex, barrel field; S1DZ, primary somatosensory cortex, dysgranular zone; S1FL, primary somatosensory cortex, forelimb region; S1Sh, primary somatosensory cortex, shoulder region; S1Tr, primary somatosensory cortex, trunk region; S1ULp, primary somatosensory cortex, upper lip region; TeA, temporal association cortex; V1, primary visual cortex; V1M, primary visual cortex, monocular area; V1B, primary visual cortex, binocular area; V2L, secondary visual cortex, lateral area; V2ML secondary visual cortex, mediolateral area; V2MM, secondary visual cortex, mediomedial area. Area is shown as a mean ± standard error of the mean (SEM). Animal numbers are in parenthesis. Statistical significances: *, *p* < 0.05 as compared to the area in the EEG cohort (Mann-Whitney U); ^#^, *p* < 0.05 as compared to the percentage of rats in the EEG cohort (ꭕ^2^ test).

## Data Availability

The data presented in this study are available on request from the corresponding author.

## Supplementary Materials

The following supporting information can be downloaded at: https://www.mdpi.com/article/10.3390/biomedicines10092295/s1. Supplementary Table S1. The distribution of the dorsoventral location of the lower tip of the anterior and posterior intracortical electrodes in sham-operated and TBI rats. Supplementary Table S2. Location of the “virtual electrode”. Summary of the locations of the anterior and posterior intracortical, and hippocampal electrodes in the MRI cohort, if implanted according to the atlas-based coordinates. Supplementary Figure S1. A schematic presentation of the MRI protocol for estimating the adjustments needed for atlas-based coordinates (See discussion for further details).
