# Supplementary material for: MRI-Guided Electrode Implantation for Chronic Intracerebral Recordings in a Rat Model of Post−Traumatic Epilepsy—Challenges and Gains
Source: Biomedicines. 2022 Sep 15;10(9):2295. doi: 10.3390/biomedicines10092295 (PMC9496327; doi:10.3390/biomedicines10092295)
Supplement: Supplementary file 1 [file biomedicines-10-02295-s001.zip › biomedicines-1881131-supplementary.pdf]

Supplementary Table S1. The distribution of the dorsoventral location of the lower tip of the anterior and posterior acortical electrodes in sham-operated and TBI rats

**EEG Cohort**	**All Cases (p<0.001)**		**Anterior Electrode (p>0.05)**		**Posterior Electrode (p>0.05)**	
	**Anterior**	**Posterior**	**Sham**	**TBI**	**Sham**	**TBI**
Layer IV						
Layer V	28% (16/57)	6% (3/52)*	36% (5/14)	26% (11/43)	7% (1/14)	5% (2/38)
Layer VI	54% (31/57)	35% (18/52)*	43% (6/14)	58% (25/43)	14% (2/14)	42% (16/38)
Corpus callosum		40% (21/52)***			57% (8/14)	34% (13/38)
External capsule	18% (10/57)		21% (3/14)	16% (7/43)		
Hippocampus						
Ventricle						
Subiculum		10% (5/52)			21% (3/14)	5% (2/38)
Cavity		10% (5/52)*				13% (5/38)
MRI cohort	All cases (p<0.01)		Anterior electrode (p>0.05)		Posterior electrode (p>0.05)	
	Anterior	Posterior	Sham	TBI	Sham	TBI
Layer IV		3% (1/40)				3% (1/29)
layer V	31% (13/42)	20% (8/40)	9% (1/31)	39% (12/31)	18% (2/7)	21% (6/29)
Layer VI	45% (19/42)	38% (15/40)	64% (7/31)	39% (12/31)	27% (3/7)	41% (12/29)
Corpus callosum	7% (3/42)	3% (1/40)	9% (1/31)	7% (2/31)		3% (1/29)
External capsule	17% (7/42)	5% (2/40)	18% (2/31)	16% (5/31)	9% (1/7)	3% (1/29)
Hippocampus		28% (11/40)***			46% (5/7)	21% (6/29)
Ventricle		5% (2/40)				7% (2/29)
Subiculum						
Cavity						

Animal numbers are in parenthesis. Abbreviations: Cavity, cortical lesion cavity. Statistical significances: *p<0.05, **p<0.01, ***p<0.001 as compared to anterior intracortical electrode (χ^2 test).

Supplementary Table S2. Location of the "virtual electrode". Summary of the locations of the anterior and posterior intracortical, and hippocampal electrodes in the MRI cohort, if implanted according to the atlas-based coordinates.

		Anterior Cortical Electrode				Hippocampal Electrode				Posterior Cortical Electrode			
		MRI-Based		Atlas-Based		MRI-Based		Atlas-Based		MRI-Based		Atlas-Based	
**Animal**	**Group**	**AP Level**	**DV Location**	**AP Level**	**DV Location (Distance from Lesion Edge)**	**AP Level**	**DV Location**	**AP Level**	**DV Location**	**AP Level**	**DV Location**	**AP Level**	**DV location (Distance from Lesion Edge)**
#1017	Sham	−0.12	Layer VI	−1.72	Layer VI	−2.4	CA3b	−3	mol	−5.52	Layer V	−7.56	Angular bundle
#1035	Sham	−0.12	Layer VI	−1.72	Layer V	−3.24	CA3c	−3	mol	−5.28	dHC	−7.56	Layer VI
#1045	Sham	−0.48	Layer VI	−1.72	Layer VI	−2.76	CA3b	−3	i-gcl	−4.2	HC-SO	−7.56	Layer VI
#1085	Sham	−0.48	Layer VI	−1.72	Layer V	−3	i-gcl	−3	Hilus	−4.44	dHC	−7.56	Layer 6
#1091	Sham	−1.32	Layer VI	−1.72	Layer V	−3.24	CA3c	−3	i-gcl	−5.2	Layer VI	−7.56	Layer VI
#1096	Sham	−1.32	External capsule	−1.72	Layer VI	−2.52	i-gcl	−3	Hilus	−4.2	HC-CA1	−7.56	Layer VI
#1107	Sham	−1.2	Layer V	−1.72	Layer V	−2.76	CA3c	−3	mol	−4.2	Layer VI	−7.56	Layer VI
#1143	Sham	−2.04	Layer VI	−1.72	Layer V	−3.36	i-gcl	−3	Hilus	−4.2	dcw	−7.56	Layer VI
#1146	Sham	−2.76	Layer VI	−1.72	Layer V	−3.6	hf	−3	i-gcl	−5.2	Layer V	−7.56	Layer VI
#1155	Sham	−3.24	Corpus callosum	−1.72	Layer V	−3.48	mol	−3	i-gcl	−4.92	Layer VI	−7.56	Layer VI
#1161	Sham	−2.04	External capsule	−1.72	Layer VI	−3.12	i-gcl	−3	mol	−3.96	dcw	−7.56	Layer VI
#1008	TBI	−0.36	Layer VI	−1.72	Ventricle	−1.08	Ventricle	−3	CA3c	−4.68	Layer VI	−7.56	Lesion cavity
#1012	TBI	0.12	Layer VI	−1.72	External capsule (medial, 0.9 mm)	−1.2	ventricle	−3	CA3c	−5.2	HC-CA1	−7.56	Lesion cavity
#1019	TBI	0	Layer V	−1.72	Layer V (anterior, 0.9 mm)	−2.76	CA3c	−3	i-gcl	−4.2	HC-CA1	−7.56	PC (medial, 0 mm)
#1024	TBI	−0.24	Corpus callosum	−1.72	External capsule (medial, 0.6 mm)	−2.76	s-gcl	−3	i-gcl	−4.68	Cingulum	−7.56	Lesion cavity
#1028	TBI	0.24	Layer VI	−1.72	Ventricle	−1.72	ventricle	−3	s-gcl	−4.36	Layer VI	−7.56	Lesion cavity
#1029	TBI	0.6	Cingulum	−1.72	Ventricle	−1.72	ventricle	−3	CA3b	−4.36	dcw	−7.56	Lesion cavity
#1031	TBI	0	Layer V	−1.72	Layer 6 (medial, 1.3 mm)	−2.76	CA3c	−3	CA3c		Not known	−7.56	Lesion cavity
#1036	TBI	−0.48	External capsule	−1.72	Ventricle	−3.12	CA3b	−3	CA3c	−5.28	Alveus	−7.56	Lesion cavity
#1038	TBI	−0.36	External capsule	−1.72	Layer VI (medial, 1.2 mm)	−2.76	Fimbria	−3	mol	−4.68	Alveus	−7.56	PC (medial, 0.7 mm)
#1043	TBI	0.6	Layer V	−1.72	Layer VI (medial, 0.9 mm)	−3	i-gcl	−3	CA3c	−3.96	Layer IV	−7.56	Lesion cavity
#1046	TBI	0.36	Layer VI	−1.72	Ventricle	−3	hf	−3	CA3c	−4.08	Layer V	−7.56	Lesion cavity
#1090	TBI	−1.08	External capsule	−1.72	Layer VI (medial, 0.8 mm)	−1.92	ventricle	−3	CA3c	−4.36	Ventricle	−7.56	PC (medial, 0.53 mm)
#1095	TBI	0	External capsule	−1.72	External capsule (medial 1 mm)	−2.28	CA3c	−3	mol	−3.96	Layer VI	−7.56	PC (medial, 0.32 mm)
#1099	TBI	−0.48	Layer VI	−1.72	Ventricle	−3.24	CA1	−3	i-gcl	−4.2	Layer VI	−7.56	PC (medial, 0.93 mm)
#1103	TBI	−0.48	Layer V	−1.72	External capsule (anterior, 0.6 mm)	−2.76	mol	−3	CA3c	−4.2	Layer V	−7.56	PC (caudal, 0.2 mm)
#1104	TBI	−1.72	Layer V	−1.72	Layer 6 (anterior 0,.6 mm)	−2.92	CA3c	−3	CA3c	−5.28	Layer V	−7.56	PC (medial, 0.73 mm)
#1105	TBI	−0.96	Layer V	−1.72	Ventricle	−3	CA3c	−3	CA3c	−4.68	Layer VI	−7.56	Lesion cavity
#1138	TBI	−1.2	Layer V	−1.72	Ventricle	−2.76	i-gcl	−3	CA3c	−3.96	Layer V	−7.56	PC (medial, 0.71 mm)
#1139	TBI	−2.04	Layer VI	−1.72	Layer VI (medial, 1.2 mm)	−3.24	i-gcl	−3	CA3c	−5.4	Layer V	−7.56	Lesion cavity
#1140	TBI	−2.76	Layer V	−1.72	Layer V (anterior, 0.5 mm)	−4.2	CA1	−3	i-gcl	−5.52	Layer VI	−7.56	PC (medial, 1.1 mm)
#1142	TBI	−1.92	Layer VI	−1.72	Ventricle	−3.84	i-gcl	−3	CA3c	−4.2	HC-SO	−7.56	Lesion cavity
#1144	TBI	−1.8	Layer VI	−1.72	Ventricle	−2.76	CA3b	−3	mol	−4.8	Ventricle	−7.56	Lesion cavity
#1145	TBI	−1.56	Layer VI	−1.72	External capsule (lateral 1.1 mm)	−3	mol	−3	i-gcl	−4.2	SO/ CA1	−7.56	PC (medial, 0.63 mm)
#1149	TBI	−3.36	External capsule	−1.72	Layer VI (medial, 1.1 mm)	−2.4	s-gcl	−3	CA3c	−4.2	Layer VI	−7.56	Lesion cavity
#1150	TBI	−2.4	Layer VI	−1.72	Ventricle	−2.76	CA3	−3	CA3c	−4.2	Layer VI	−7.56	Lesion cavity
#1152	TBI	−2.52	Layer V	−1.72	Layer V (anterior, 0.5 mm)	−3.36	hf	−3	mol	−4.68	Layer V	−7.56	PC (medial, 0.91 mm)
#1153	TBI	−1.32	Layer VI	−1.72	Ventricle	−2.76	CA3c	−3	CA3c	−4.2	Layer VI	−7.56	Lesion cavity
#1154	TBI	−2.16	Layer V	−1.72	Ventricle	−3.24	CA3c	−3	CA3c	−5.4	Layer VI	−7.56	Lesion cavity
#1156	TBI	−3.48	Layer VI	−1.72	Ventricle	−3	GCL	−3	CA3c	−4.68	Layer VI	−7.56	PC (medial, 0.28 mm)
#1158	TBI	−2.76	Layer V	−1.72	External capsule (medial, 0.8 mm)	−3.36	s-gcl	−3	Hilus	−4.2	Layer VI	−7.56	Lesion cavity
#1159	TBI	−1.56	Layer V	−1.72	Ventricle	−3	CA3c	−3	CA3c		Not known	−7.56	PC (medial, 1.3mm)

Abbreviations: AP, anteroposterior; CA3, CA3 (CA3b, CA3c) subfield of the hippocampus; CA1, CA1 subfield of the hippocampus; cg, cingulum; DV, dorsoventral; dHC, dorsal hippocampus; dcw, deep cerebral white matter; HC, hippocampus; hf, hippocampal fissure; gcl, granule cell layer (s-gcl, suprapyramidal blade, i-gcl, infrapyramidal blade); mol, molecular layer; PC, perilesional cortex; SO, stratum oriens;

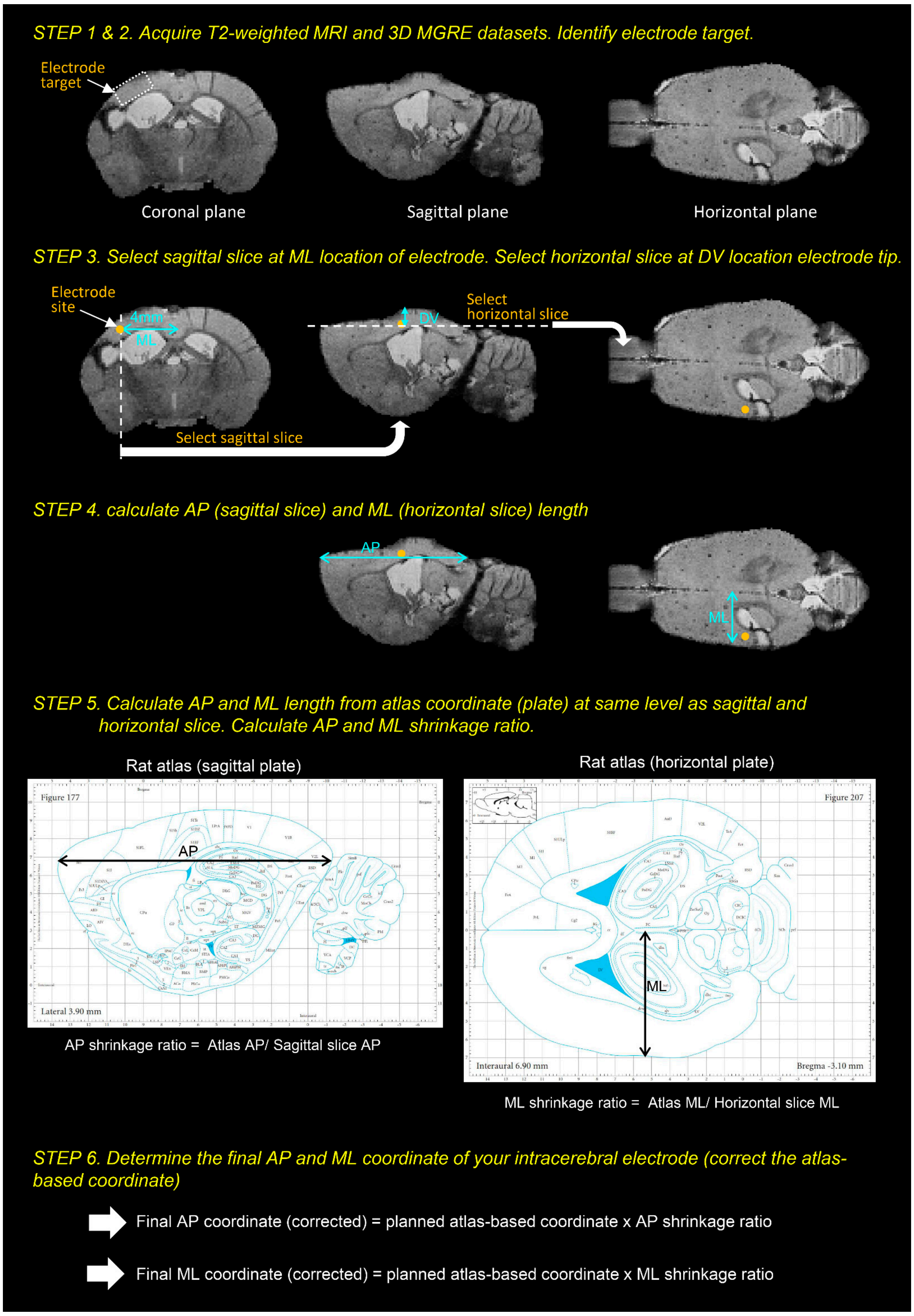

Supplementary Figure S1. A schematic presentation of the MRI protocol for estimating the adjustments needed for atlas-based coordinates (See discussion for further details.
